# A Novel *Seimatosporium* and Other *Sporocadaceae* Species Associated with Grapevine Trunk Diseases in Cyprus

**DOI:** 10.3390/plants11202733

**Published:** 2022-10-16

**Authors:** Loukas I. Kanetis, Demetris Taliadoros, Georgios Makris, Michalis Christoforou

**Affiliations:** 1 Department of Agricultural Sciences, Biotechnology and Food Science, Cyprus University of Technology, Limassol 3036, Cyprus; 2 Environmental Genomics Group, Max Planck Institute for Evolutionary Biology, 24306 Plön, Germany; 3 Department of Biology, Christian-Albrechts University of Kiel, 24118 Kiel, Germany

**Keywords:** Cyprus, grapevine trunk diseases, *Seimatosporium*, *Sporocadaceae*, *Sporocadus*, *Vitis vinifera*

## Abstract

Besides well-known grapevine trunk disease (GTD)-related pathogens, there is an increased interest in wood-colonizing fungi that infect grapevines. During 2017–2018, a survey was conducted in Cyprus and wood samples were collected from vines exhibiting typical GTD symptoms. Based on morphological and multilocus phylogenetic analyses (ITS, LSU, *bt2*, *tef1-a*), four species in the *Sporocadaceae* family were described and typified; two in the genus of *Seimatosporium*: *Seim. cyprium* sp. nov. and *Seim. vitis-viniferae* and two in *Sporocadus*: *Spo. kurdistanicus* and *Spo. rosigena*. The teleomorph of *Seim. cyprium* sp. nov. was also described. Pathogenicity trials with representative isolates of each species were performed on woody stems of two-year-old potted grapevines for 12 months under field conditions. All isolates were pathogenic, causing dark brown to black vascular discoloration, extending upward and downward from the inoculation point. *Sporocadus* isolates were significantly more aggressive than *Seimatosporium* with lesion lengths ranging from 9.24 to 6.90 and 4.13 to 4.00 cm, respectively. Successful re-isolations were also evident for all species and isolates. *Seim. cyprium* sp. nov. is a newly described species, while *Spo. kurdistanicus* and *Spo. rosigena* are reported for the first time in Europe on *Vitis vinifera*, suggesting the potential role of *Sporocadaceae* in the GTDs complex.

## 1. Introduction

Grapevine trunk diseases (GTDs) form an aggregate of fungal diseases that are currently considered the most destructive biotic factor of grapevines globally [1,2,3,4]. Multifaceted adverse effects due to GTDs include reduced longevity and profitable vineyard lifespan [5], cumulative yield losses, increased costs due to applied management practices, and premature replanting of severely affected vineyards [6,7]. Although long considered a deteriorating factor of viticulture, GTDs’ incidence and subsequent consequences have spiked along the last three decades, mainly due to the circulation of potentially contaminated planting material, an industry shift towards cultivation systems that render vines more prone to wood infections, as well as the lack of effective plant protection products [6,8,9,10].

To date, over 140 fungal species (predominately ascomycetous) belonging to 35 genera have been reported to be associated with GTDs worldwide, while numerous grapevine microbiome studies enrich our knowledge on species involved with GTDs; however, the degree of involvement of many GTD-related species remains elusive [6,11,12,13].

Several fungi within the family of *Sporocadaceae* (*Xylariales*, *Sordariomycetes*) have been reported as saprophytes, endophytes, and plant pathogens in a wide host range [14,15,16,17,18,19]. More specifically, species within *Neopestalotiopsis* Maharachch., K.D. Hyde and Crous, *Pestalotiopsis* Steyaert, *Seimatosporium* Corda, *Sporocadus* Corda, and *Truncatella* Steyaert have been recently found in association with *Vitis vinifera,* either as part of its mycobiome [12,20,21,22] or as components of the GTD complex [13,16,23,24,25,26,27,28,29]. The *Sporocadaceae* family was revived by Jaklitsch et al. (2016) to accommodate coelomycetous fungi, also known as pestalotioid, characterized by multi-septate conidia, bearing appendages at one or both end-cells [30]. Due to the restricted delineation capabilities of morphological features, phylogenetic studies based on multi-locus sequence data have contributed to address controversial ambiguities in *Sporocadaceae* [30,31,32,33]. More recently, an extensive multigene phylogenetic study combined with morphological data on appendaged coelomycetous by Liu et al. (2019) placed them in *Sporocadaceae* and recognized 30 monophyletic genera, including *Seimatosporium* and *Sporocadus* also [34].

The genus *Seimatosporium* was erected with *Seim. rosae* Corda as the type species and is characterized by septate and branched conidiophores with discrete integrated conidiogenous cells grown within acervuli or pycnidia that produce mostly 3-septate (3 to 6) mostly fusiform, ovoid to falcate conidia with brown-colored zed median cells with or without a single apical (centric) and basal (excentric) appendage [34]. *Seimatosporium* and *Sporocadus* were initially synonymized [35]; however, the latter was recently resurrected as a distinct genus and lectotypified on *Spo. lichenicola* Corda, Icon. fung. (Prague); accommodating species with fusoid, ellipsoidal to obovoid, multi-septate (1–7), mostly 3-septate conidia, bearing colorless obconic basal and apical cells with a truncate base and a round apex, respectively, and pale brown to brown median cell [26,34,36]. Notably, the conidia of *Sporocadus* species are predominately non-appendaged apart from *Spo. trimorphus* and *Spo. rosarum* [34].

Currently, nine *Seimatosporium* and five *Sporocadus* species have been found in association with *V*. *vinifera* worldwide. More specifically, *Seim. botan* Sat. Hatak. and Y. Harada, *Seim. hysterioides* (Fuckel) Brockmann, *Seim. lonicerae* (Cooke) Shoemaker, *Seim. luteosporum* D.P. Lawr. and Travadon, *Seim. marivanicum* Abdollahz., Nahvi M. and Khaledi E., *Seim. parasiticum* (Dearn. and House) Shoemaker, *Seim. vitifusiforme* D.P. Lawr. and Travadon, *Seim. vitis* Y.P. Xiao, Camporesi and K.D. Hyde, and *Seim. vitis-viniferae* F. Liu, L. Cai and Crous [12,13,26,34,37,38] have been reported in Australia, Chile, Iran, Italy, Hungary, USA. Although *Seim. fusisporum* H. J. Swart and D.A. Griffiths [24] and *Seim. macrospermum* (Berk. and Broome) B. Sutton have been also associated with grapevine, the former has been re-evaluated to a sister clade and classified as *Allelochaeta fusispora* (H. J. Swart and D. A. Griffiths) Crous [39], while the latter, given the absence of conidial appendages it has been moved to *Sporocadus*, as *Spo. macrospermus* (Berk. and Broome) M. Morelet [34,40], along with *Spo. kurdistanicus* Abdollahz., Nahvi M. and Khaledi E., *Spo. lichenicola*, *Spo. rhododendri* (Schwein.) M. Morelet, and *Spo. rosigena* F. Liu, L. Cai and Crous [26,27,34], that have also been associated with *V. vinifera*. However, limited information is available regarding their pathogenic potential, mainly reported as endophytic saprobes of grapevine and other hosts [11,12,18,41,42,43].

Viticulture is essential for the economy and rural development of Cyprus, covering an acreage of 6790 ha with an annual yield of 23,570 tons [44]. However, the severity of GTDs, as well as the identity of the implicated microorganisms and their pathogenic status remains undescribed and needs to be studied in the country [45]. Considering the numerous *Sporocadaceae* isolations from grapevines exhibiting GTD symptoms in Cyprus, combined with multiple reports around the world, our objectives are: (a) to characterize *Sporocadaceae* species diversity in the local vineyards, based on morphological and molecular analyses, and (b) to evaluate their pathogenic potential on grapevines.

## 2. Results

### 2.1. Molecular Identification and Phylogenetic Analyses

The best-fit nucleotide substitution model per locus based on the Bayesian Information Criterion (BIC) were: Kimura-2 parameter, also allowing for a proportion of invariant sites (I) and gamma distributed rates (G) (K2P + I + G) for the internal transcribed spacer regions and the 5.8 gene (ITS); Tamura-Nei parameter with I and G4 (TNe + I + G) for partial regions of the ribosomal large subunit (LSU); three substitution site model with empirical-based frequencies (F) and I (TPM3 + F + I) for the translation elongation factor 1-a (*tef1-a*) gene, and Hasegawa-Kishino-Yano (HKY) with F, I, and G4 (HKY + F + I + G) for the β-tubulin (*tub2*) gene. Phylogenetic trees of single-locus alignments obtained using the models suggested that four major clades belonging to the genera *Allelochaeta*, *Sarcostroma*, *Seimatosporium*, and *Sporocadus* were obtained for *tub2* and *tef1-a*. More specifically, the *tef1-a* tree was the only one that enabled the separation of all species with high bootstrap values (Supplementary Figure S1). For *tub2*, all species could be separated, except *Seim. vitifusiforme* (CBS 142600) and *Seim. marivanicum* (CBS 143781) that were clustered in the same clade (Supplementary Figure S2). The ITS and LSU trees could not properly resolve at the species and/or genus level, due to high levels of nucleotide similarities in these regions (Supplementary Figures S3 and S4).

**Figure 1:**
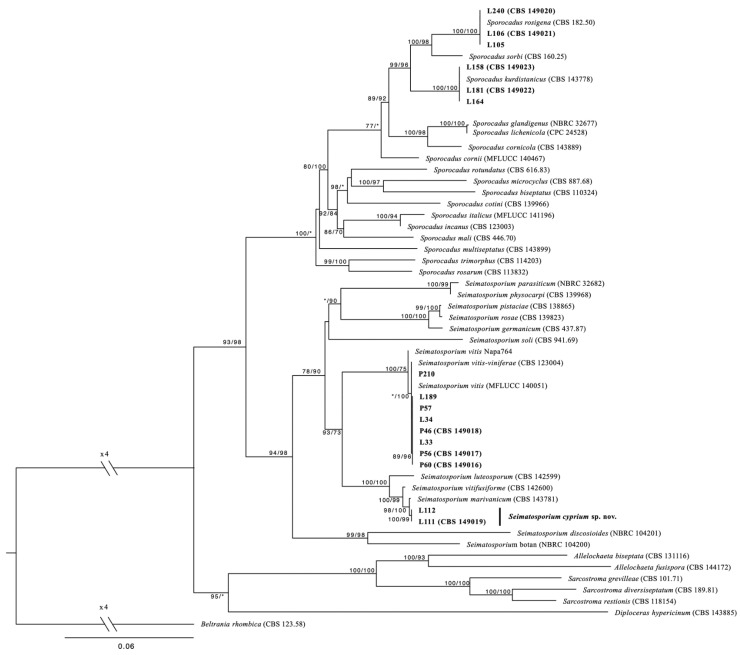
Phylogenetic tree (log-likelihood: −15,895.468) resulting from the combined analysis of ITS, LSU, *tub2*, and *tef1* sequence data from 54 *Sporocadaceae*. Numbers represent maximum-likelihood and maximum-parsimony bootstrap values, respectively. Values represented by an asterisk were less than 70%. Scale bar represents the expected number of substitutions per site. The tree was rooted to *Beltrania rhombica* (CBS 123.58).

Sequence alignment of the of the four loci (ITS, LSU, *tub2*, and *tef1-a*) resulted in a 2375-character dataset, with 1539 being constant, 616 were parsimony informative, and 229 were variable and parsimony uninformative. After 100 ratchet iterations, the maximum parsimony (MP) analysis produced five equally most-parsimonious trees of 696 steps and a consistency index (CI) and retention index (RI) of 0.459716 and 0.754707, respectively. For the maximum likelihood (ML) analysis the ModelFinder algorithm of IQtree indicated a transition model with equal bases frequencies (TIM2e) with gamma distributed rates, allowing a proportion of invariant sites (TIM2e + I + G) nucleotide substitution model as the best fit for the concatenated dataset. ML and MP analyses revealed that all four *Sporocadaceae* species recovered from symptomatic grapevine cankers in Cyprus and are strongly supported (100 and ≥96%, respectively) as independent phylogenetic lineages (Figure 1).

### 2.2. Morphological Description

The color of *Sporocadaceae* colonies ranged from white to grayish and from light brown to light sepia on PDA and MEA, while on OA, they were from grayish white to light sienna or brown. Characters used to distinguish the species included the relative cell lengths of conidia, conidial septation, and the appendage morphology. Based on microscopic observations, all isolates produced septate conidia, which matched the descriptions for *Sporocadaceae*-like asexual forms. However, *Sporocadaceae* isolates were separated into two different groups. The first consisted of isolates (n = 6) showing mostly 3-septate, obovoid, thick-walled, pale brown to brown conidia that lacked appendages at both the basal and apical ends. These features matched those described earlier for the genus *Sporocadus* [34]. The second group (n = 10) included isolates with fusoid, pale brown, mostly 3- up to 6-septate conidia, bearing basal and apical (sometimes absent appendages, which resembled those of the genus *Seimatosporium* [34]. There were significant differences in conidial length (*p* [*F*_(1, 209)_ > 1058] < 0.0001) and width (*p* [*F*_(1, 209)_ > 1208] < 0.0001) between *Sporocadus* species, with *Spo. kurdistanicus* being on average longer and wider as compared to *Spo. rosigena*. Similarly, *Seim. vitis-viniferae* produced, on average, smaller conidia to *Seim. cyprium*, with significant differences in their conidial length (*p* [*F*(_1, 121)_ > 57.41] < 0.0001) and width (*p* [*F*_(1, 121)_ > 88.15] < 0.0001). Homothallic crosses were performed to induce sexual reproductive structures in obtained *Sporocadaceae* isolates. *Seim. cyprium* was the only species that produced abundant perithecia immersed on grapevine wood segments bearing eight uniseriate, single-septate, ascospores per ascus.

### 2.3. Effect of Temperature on Mycelial Growth

All *Sporocadaceae* isolates were able to grow at all tested temperatures and an analyses of variance (ANOVA) indicated no significant differences (*p* < 0.05) of the mycelial growth among the experiments, thus the data were combined. The relationship between mycelial growth at different temperatures was best described by a cubic response model (*y* = *a*T^3^ + *b*T^2^ + *c*T + *d*). All regression coefficients were significantly different (*p* < 0.01), and the coefficients of determination (*R*^2^) ranged from 0.89 to 0.99 (Table 1). Based on the adjusted models derived per isolate, the optimum temperatures of mycelial growth on PDA ranged from 20.3 to 24.1 °C with significant differences detected among them (Table 1). More specifically, optimum temperature ≥ 23 °C included only *Seim. cyprium* isolates. Isolates with an optimum temperature between 22 and 21 °C were *Seim. vitis-vineferae* and *Spo*. *rosigena* isolates, as well as the *Spo. kurdistanicus* isolate P158. The only species that had an optimum temperature < 21 °C was the *Spo. kurdistanicus* L181. The maximum mycelial growth rates also differed significantly (mean = 4.4; median = 4.4). More specifically, all isolates had a maximum growth rate > 4 mm/day, ranging from 4.1 to 4.8, except L181 with < 4 mm/day (Table 1). Furthermore, maximum growth rates on MEA cultures ranged from 2.6 to 5 mm/day (mean = 4.3; median = 4.9), with isolates L181 and the *Seim. cyprium* L111 bearing the highest and lowest values, respectively, and optimum growth temperatures 20.4–24.4 °C (data not shown). The highest average growth rate for *Sporocadaceae* isolates was evident on OA ranging from 4.4 to 5.9 mm/day (mean = 5.3; median = 5.5), with P60 and L34 being the slowest- and fastest-growing isolates, respectively, and optimum growth temperatures from 21.4 to 24.3 °C (data not shown).

Morphological comparisons, coupled with phylogenetic analyses of the ITS, LSU, *bt2*, and *tef1-a* dataset, identified four distinct and strongly supported lineages, one of which has no apparent species name. Thus, we propose the following new species name to properly circumscribe and typify this unique taxon.

**Figure 2:**
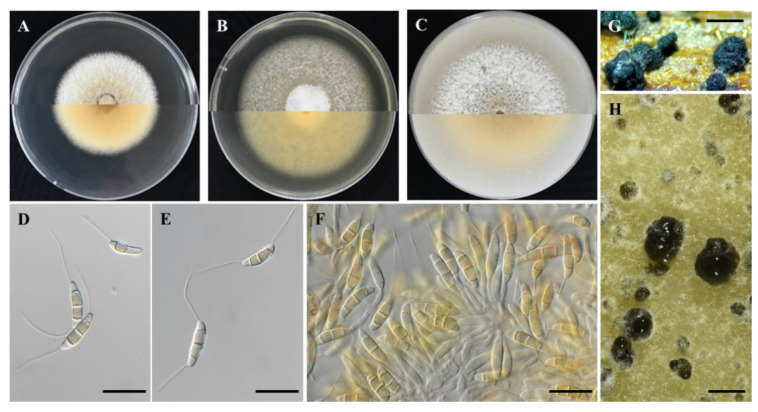
*Seimatosporium cyprium* isolate L111 (CBS 149019). (**A**–**C**) Colonies cultured on PDA, MEA and OA, respectively, at 25 °C in the dark for 14 days (top: above and bottom: reverse). (**D**,**E**) Septate conidia with long basal and apical appendages. (**E**,**F**) Conidiophores, conidiogenous cells and conidia. (**G**) Superficial conidiomata on autoclaved grapevine wood segments. (**H**) Conidiomata on OA. Scale bars: 20 μm (**D**–**F**) and 500 μm (**G**,**H**).

**Figure 3:**
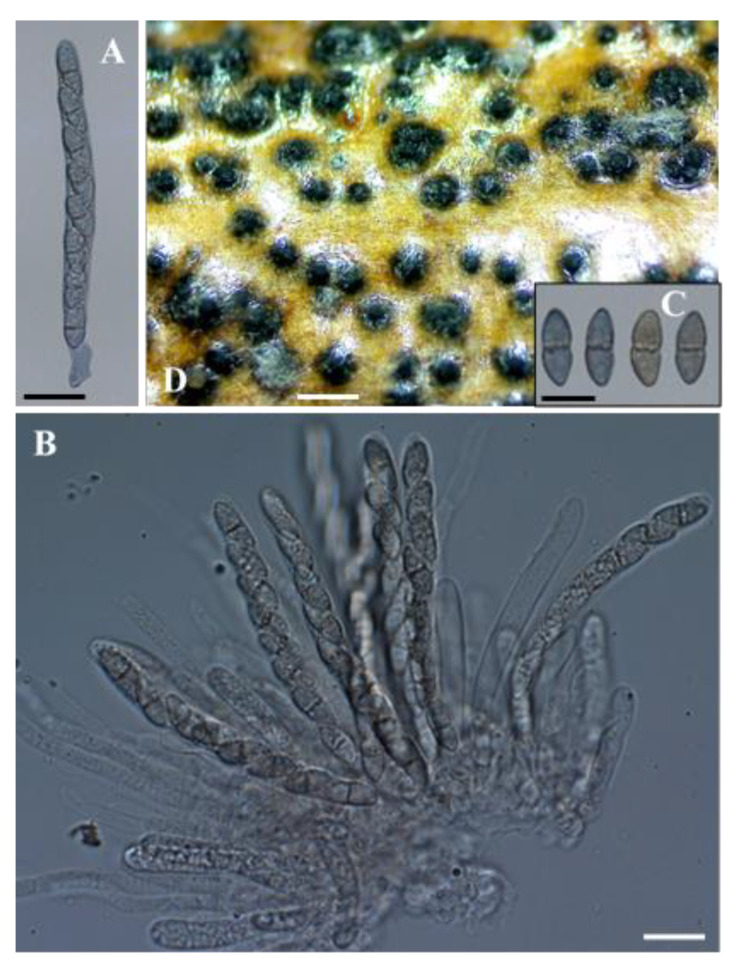
*Seimatosporium cyprium* isolate L111 (CBS 149019). (**A**,**B**) Cylindrical, 8-spored asci. (**C**) Fusiform, 2-celled ascospores. (**D**) Semi-immersed perithecia on autoclaved grapevine wood at 10 °C. Scale bars: 20 μm (**A**–**C**) and 500 μm (**D**).

***Seimatosporium cyprium*** Kanetis L. and Makris G., sp. nov., Figure 2 and Figure 3.

MycoBank number: MB 845835.

Etymology: Epithet refers to the country where the species was first found.

Type: CYPRUS: Limassol Province, Chandria; 34.940583°N, 32.997139°E, 1200 meters above sea level (masl); isolated from wood canker of *V. vinifera*, June 2017, Kanetis L. (holotype L111; ex-type CBS 149019). GenBank accession numbers: ON680684, ITS; ON705769, LSU; ON695856, *bt2*; ON863790, *tef1-a*.

Description: Sexual morph: *Ascomata* perithecial, stromatic, appearing on the surface of grapevine wood segments, dark circular patches, 198–441 μm (av. = 300 ± 63.8 μm, n = 30) diam., solitary, slightly immersed or directly below the wood epidermis, depressed globose, membranous, black, with a centrical ostiole. *Asci* 80.8–109.1 × 6.5–9.5 μm (av. = 98.6 ± 8.47 × 8.2 ± 0.91 μm (n = 20), 8-spored, unitunicate, cylindrical, straight, or slightly curved, pedicellate, apically rounded. *Ascospores* 11.5–16.9 × 5.1–6.8 μm (14.5 ± 1.19 × 5.9 ± 0.43 μm, n = 30), uniseriate, or overlapping uniseriate, hyaline, fusiform to ellipsoidal, hyaline, 1-septate, smooth-walled. Asexual morph: *Conidiomata* in pure cultures, acervular, dark brown, black, scattered, erumpent, superficial or semi-immersed; on grapevine wood segments 265–774 μm (501 ± 118 μm, n = 30) in diameter; *conidiophores* branched, cylindrical or reduced to conidiogenous cells, colorless, smooth; *conidiogenous cells* discrete, mostly cylindrical, slightly curved, colorless, smooth, 10.1–26.4 × 1.4–2.2 μm (16.3 ± 4.15 × 1.8 ± 0.28 μm; n = 15). *Conidia* straight or slightly curved, 3-septate, wall smooth, 15.7–23.1 × 3.9–5.3 μm (av. = 18.5 ± 1.61 × 4.5 ± 0.32 μm; n = 50), bearing appendages; basal cell obconic, with a truncate base, subcylindrical, colorless, 2.7–5.5 μm (av. = 4.3 ± 0.64 μm) long; two median cells, pale brown, ± equal, each 3.9–6.8 μm (av. = 5.2 ± 0.58 μm) long; apical cell obtuse or conical, colorless, 3.1–6.1 μm (av. = 4.1 ± 0.61 μm) long, apical appendage single, unbranched, 5.8–32.2 μm (av. = 19.6 ± 5.05 μm) long; basal appendage single or rarely dual, unbranched or rarely branched, excentric, 2.6–29.0 μm (av. = 16.5 ± 6.32 μm) long; mean conidium length/width ratio = 4.1:1.

Colony of *Seim. cyprium* isolate L111 (CBS 149019) on PDA growing, slightly raised, greyish white with smooth wooly margin, reaching 53.5 mm in diameter after 14 days at 25 °C in darkness. Colony on MEA slightly raised, wooly white with smooth wooly margin, reaching 64.75 mm in diameter after 14 days at 25 °C in darkness. Colony on OA raised, wooly light sienna to white with smooth margin, reaching 69.75 mm in diameter after 14 days at 25 °C in darkness. The optimum temperature of mycelial growth on PDA was estimated at 23.8 °C with a growth rate of 4.4 mm/day.

Additional specimen examined: CYPRUS: Limassol Province, Kyperounda, 34.944722°N, 32.98725°E, 1,300 masl; isolated from wood canker of *V. vinifera*, June 2017, L. Kanetis (L112). GenBank accession numbers: OΝ695889, ITS; ON692404, LSU; ON695848, *bt2*; ON863791, *tef1-a*.

Ecology and host characteristics: Isolates L111 (=CBS 149019) and L112 belonging to the present species were recovered from vines of the indigenous wine grape cv. Mavro. Isolate L111 was co-isolated with *Phaeomoniella chlamydospora*.

Distribution: Cyprus.

Notes: Based on multi-locus phylogenetic analyses *Seim. cyprium* formed a distinct, highly supported clade that is closely located to *Seim. marivanicum* (ML/MP = 100/99), *Seim. vitifusiforme* (ML/MP = 100/99), and *Seim. luteosporum* (ML/MP = 100/100). *Seim. cyprium* differs from *Seim. marivanicum* in ITS (one substitution), *tub2* (two substitutions), and *tef1-a* (one insertion or deletion), while their LSU data are identical, however the species can be distinguished in the *tef1-a* phylogram; with a total of four PWD. Despite their high sequence similarities, both species have distinct morphological differences in terms of conidial dimensions and septation, with a mean length/width ratio of 4.1 vs. 5(-6), septa number of 3 vs. 3(-6), and media cell length of 5.2 ± 0.58 vs. 8 ± 0.7 μm for *Seim. cyprium* and *Seim. marivanicum*, respectively. In addition, the apical appendages of *Seim. cyprium* are distinctly longer compared to the ex-type of *Seim. marivanicum* (Table 2). Similarly, phylogenetic analyses and estimates of evolutionary divergence between concatenated sequences used herein, support strong species differentiation (ML/MP = 99/100) of *Seim. pistaciae* (CBS 138865) and *Seim. rosae* (CBS 139823), given that they exhibit three PWD and discrete morphological characteristics (Supplementary Table S1; Figure 1) [34].

*Seim. luteosporum* has significant sequence data differences in ITS (two substitutions and two insertions or deletions), LSU (four substitutions), *tub2* (eleven substitutions and one deletion or insertion), and *tef1-a* (twelve substitutions and seven deletions or insertions) from *Seim. cyprium* and they could be clearly separated in all the single locus phylogenies, except ITS. However, *Seim. cyprium* cannot be clearly differentiated from *Seim. luteosporum* by conidial characteristics (Table 2). The LSU sequences of *Seim. cyprium* and *Seim. vitifusiforme* are identical, but they differ in ITS (one substitution), *tub2* (one substitution) and *tef1-a* (five substitutions) and can be clearly distinguished only in the *tef1-a* phylogram. The conidial characteristics of both species are also discrete (Table 2). Unlike *Seim. cyprium*, no teleomorph has been currently reported for *Seim. luteosporum*, *Seim. marivanicum*, and *Seim. vitifusiforme*.

**Figure 4:**
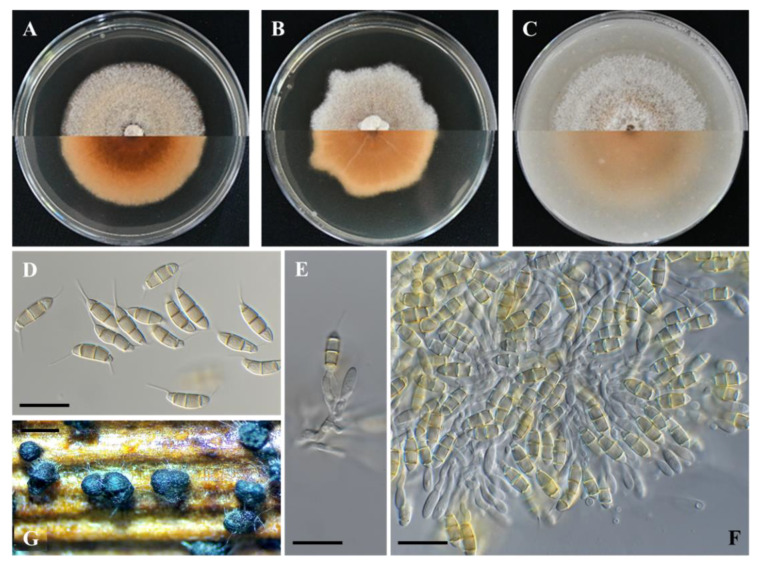
*Seimatosporium vitis-viniferae* isolate P60 (CBS 149016). (**A**–**C**) Colonies cultured on PDA, MEA, and OA, respectively, at 25 °C in the dark for 14 days (top: above and bottom: reverse). (**D**) Septate conidia with basal and apical appendages. (**E**,**F**) Septate conidiophores, conidiogenous cells and conidia. (**G**) Conidiomata on autoclaved grapevine wood segments. Scale bars: 20 μm (**D**–**F**) and 500 μm (**G**).

***Seimatosporium vitis-viniferae*** F. Liu, L. Cai and Crous, Figure 4.

MycoBank number: MB 828397.

Description: Sexual morph: unknown. Asexual morph: *Conidiomata* on pure cultures acervular, dark brown to black, scattered, semi-immersed or superficial; on grapevine wood segments 187–546 μm (359 ± 96 μm; n = 30) in diameter; *conidiophores* septate, mostly branched, sometimes reduced to conidiogenous cells, colorless, smooth. *Conidiogenous cells* discrete or integrated, cylindrical, variable in size, 8.2–40.2 × 1.3–2.7 μm (av. = 18.3 ± 7.39 × 1.9 ± 0.33 μm; n = 15). *Conidia* fusoid, straight, mostly 3- up to 5-septate, smooth wall, 13.5–18.6 × 4.6–5.8 μm (av. = 16.0 ± 1.35 × 5.2 ± 0.28 μm; n = 50); bearing appendages; basal cell obconic with a truncate base, subcylindrical, colorless or similar to that of median cells, 1.8–3.8 μm (av. = 2.9 ± 0.46 μm) long; two median cells, ± equal, each 3.7–6.1 μm (av. = 4.9 ± 0.51) long; apical cell obtuse or conical, colorless, sometimes similar to that of median cells, 2.2–4.5 μm (av. = 3.4 ± 0.64 μm) long; apical appendage lacking or, when present, single, unbranched, 2.5–24.1 μm (av. = 11.2 ± 2.5) long; basal appendage single, unbranched, excentric, 3.7–19.7 μm (10.7 ± 4.67 μm) long; mean conidium length/width ratio = 3.1:1.

Colony of *Seim. vitis-viniferae* isolate P60 (CBS 149016) on PDA, slightly raised, wooly, light brown with smooth off-white margin, reaching 48 mm in diameter after 14 days at 25 °C in darkness. Colony on MEA slow-growing, slightly raised, wooly, light brown with undulate wooly, off-white margin, reaching 52.75 mm in diameter after 14 days at 25 °C in darkness. Colony on OA, fast-growing, slightly raised brown and light brown colony with smooth wooly, off-white margin, reaching 62.75 mm in diameter after 14 days at 25 °C in darkness.

Ecology and host characteristics: Eight isolates P46 (=CBS 149018), P56 (=CBS 149017), P57, P60 (=CBS 149016), P210, L33, L34, and L189 belonging to the present species were recovered from vines of the indigenous wine grape cvs. Mavro, Promara, and Xynisteri. All were single isolations from collected wood samples of symptomatic vines, except L33, L189, and P57 that were co-isolated with *Ph. chlamydospora* and *Phaeoacremonium minimum*.

Distribution: Cyprus, Iran, Italy, New Zealand, and Spain (*V. vinifera*).

Notes: Pronounced disparities in conidial size and characteristics can distinguish *Seim. vitis* and *Seim. vitis-viniferae*. More specifically, conidia of *Seim. vitis* ex-type specimen (MFLUCC 14-0051) [31] are reported to be larger than the conidia of the relevant *Seim. vitis-viniferae* specimen (CBS 123004; Table 2) [34]. Furthermore, the same authors report that *Seim. vitis* bears 3-septate conidia with basal appendages, while *Seim. vitis-viniferae* may produce 3-6-septate and appendaged conidia basally or at both ends (Table 2). In the present study, conidial characteristics of obtained isolates matched with those attributed to *Seim. vitis-viniferae*. However, the abovementioned distinct morphological differences are not supported by phylogenetic analyses of the available sequence data. The only available sequences of the *Seim. vitis* ex-type (ITS and LSU) are identical to the *Seim. vitis-viniferae* ex-type and the results are not different when other available datasets of ITS and LSU sequences are compared. Minor differences were evident when the *tub2* sequence data obtained in this study (n = 8) and all others currently available in the NCBI database (n = 23) of *Seim. vitis* and *Seim. vitis-viniferae* were analyzed. The dissimilarities found (two substitutions and three insertions or deletions) are not consistently represented among the species, suggesting that the *tub2* locus is not informative for species separation. Regarding the *tef1-a* locus, eight substitutions and two insertions or deletions are found within the same set of sequence data. A single, consistent nucleotide substitution in position 257 (T for *Seim. vitis* and to C for *Seim. vitis-viniferae* isolates) was detected, while all other nucleotide differences appear to be variably distributed between the species (Supplementary Figure S5). In conclusion, the available sequence data (ITS, LSU, *bt2*, and *tef1-a*) cannot strongly support differentiation of these two species.

**Figure 5:**
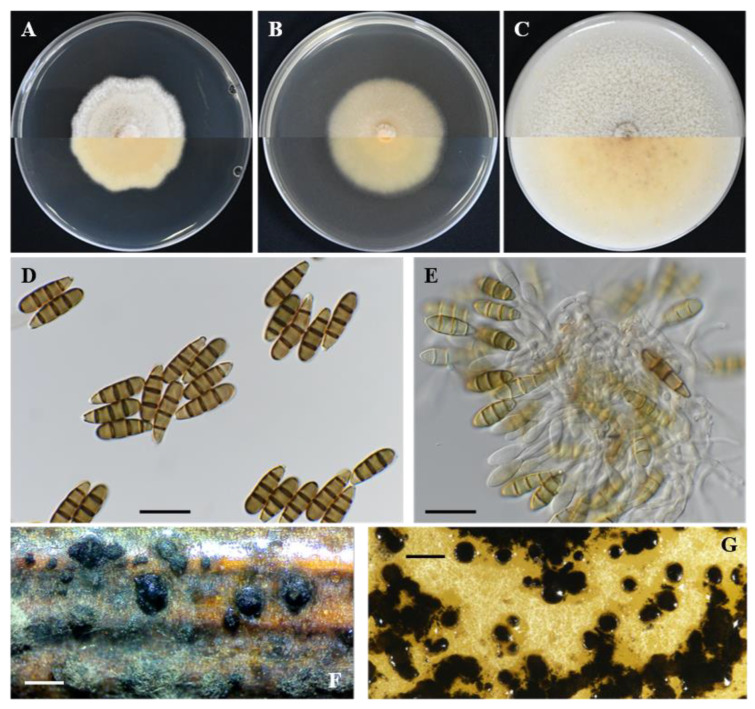
*Sporocadus kurdistanicus* isolate L181 (CBS 149022). (**A**–**C**) Colonies cultured on PDA, MEA, and OA, respectively, at 25 °C in the dark for 14 days (top: above and bottom: reverse). (**D**) Septate conidia lacking appendages. (**E**) Conidiophores, conidiogenous cells and conidia. (**F**,**G**) Conidiomata on autoclaved grapevine wood segments and OA, respectively. Scale bars: 20 μm (**D**–**E**) and 500 μm (**F**–**G**).

***Sporocadus kurdistanicus*** Abdollahz., Nahvi M. and Khaledi E., Figure 5.

MycoBank number: MB 838233.

Description: Sexual morph: unknown. Asexual morph: *Conidiomata* acervular, black, scattered, erumpent, semi-immersed; on grapevine wood segments, 210–636 μm (av. = 386 ± 89 μm; n = 30) in diameter; *conidiophores* septate, branched, sometimes reduced to conidiogenous cells, cylindrical, colorless, smooth. *Conidiogenous cells* discrete, mostly cylindrical, 16.3–50.9 × 2.3–4.6 μm (av. = 31.9 ± 8.88 × 2.9 ± 0.51 μm; n = 15). *Conidia* ellipsoid, straight or slightly curved, pale brown, and becoming mid-brown when mature, 3-septate, wall smooth, 20.6–30.1 × 7.5–10.0 μm (av. = 24.2 ± 1.88 × 8.8 ± 0.50 μm; n = 50), lacking appendages; basal cell obconic, acute or blunt base, occasionally with a narrow truncate base, pale brown or concolorous with median cells, 4.0–7.6 μm (av. = 5.4 ± 0.68 μm) long; two median cells, cylindrical to doliiform, hyaline to pale brown, and becoming mid-brown when mature, ± equal length, each 4.8–7.9 μm (av. = 6.0 ± 0.65 μm) long; apical cell conic with round apex, concolorous with the median cells, 5.7–8.7 μm (6.9 ± 0.75 μm) long; mean conidium length/width ratio = 2.8:1.

The colony of *Spo*. *kurdistanicus* isolate L181 (CBS 149022) on PDA growing, slightly raised, white with smooth, wooly, smooth margin, reaching 44 mm in diameter after 14 days at 25 °C in darkness. Colony on MEA growing, slightly raised, wooly, white with wooly, smooth margin, reaching 44 mm in diameter after 14 days at 25 °C in darkness. Colony on OA, fast-growing, slightly raised, greyish-white with wooly, smooth margin, reaching 77.75 mm in diameter after 14 days at 25 °C in darkness.

Ecology and host characteristics: Isolates L158 (=CBS 149023), L164, and L181 (= CBS 149022) belonging to the present species were recovered from vines of the indigenous wine grape cv. Xynisteri. Isolates L105 and L181 were co-isolated with *Ph. chlamydospora,* while L164 was co-isolated with *P. minimum*.

Distribution: Cyprus and Iran on *V. vinifera.*

Notes: Multigene, as well as single locus phylogeny of LSU, *bt2*, and *tef1-a* cluster ex-type and *Spo. kurdistanicus* isolates were collected in this study in a highly supported clade. Spore dimensions of isolates collected in this study are in the same range to the ex-type isolate from Iran [26], sharing similar characteristics (Table 2).

**Figure 6:**
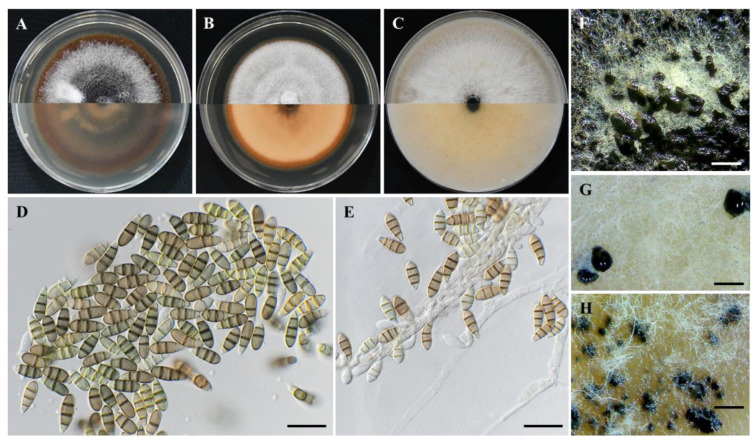
*Sporocadus rosigena* isolate L106 (CBS 149021). (**A–C**) Colonies cultured on PDA, MEA and OA, respectively, at 25 °C in the dark for 14 days (top: above and bottom: reverse). (**D**) Septate conidia lacking appendages. (**E**) Conidiophores, conidiogenous cells and conidia. (**F–H**) Conidiomata on PDA, OA, and MEA, respectively. Scale bars: 20 μm (**D**,**F**) and 500 μm (**F**–**H**).

***Sporocadus rosigena*** F. Liu, L. Cai and Crous, Figure 6.

MycoBank number: MB 828418.

Description: Sexual morph: unknown. Asexual form: *Conidiomata* black, acervular, scattered, erumpent, semi-immersed or immersed; on grapevine wood segments 349–1444 μm (av. = 805 ± 295 μm, n = 30) in diameter; *conidiophores* septate, branched, often reduced to conidiogenous cells, colorless, smooth. *Conidiogenous cells* discrete or integrated, mostly cylindrical, 15.8–41.6 × 1.6–3.2 μm (av. = 26.8 ± 7.61 × 2.4 ± 0.45 μm; n = 15). *Conidia* obovoid, ellipsoid, or subcylindrical, mostly 3- occasionally 2-septate, wall smooth, 13.2–17.9 × 5.3-6.8 μm (av. = 15.4 ± 0.99 × 6.2 ± 0.40 μm; n = 50), lacking appendages; basal cell obconic with acute or blunt base, hyaline to pale brown, or concolorous with median cells, 2.9–4.7 μm (av. = 3.7 ± 0.45 μm) long; two median cells, short-cylindrical to doliiform, hyaline or pale brown, and becoming mid-brown when mature, ± equal length, each 2.8–4.6 μm (av. = 3.7 ± 0.42 μm) long; apical cell conic, concolorous with the median cells, 3.8–7.3 μm (av. = 4.7 ± 0.86 μm) long; mean conidium length/width ratio = 2.5:1.

Colony of *Spo. rosigena* isolate L106 (CBS 149021) on PDA fast-growing, flat, dark mouse gray to light sepia with smooth margin, reaching 58.25 mm in diameter after 14 days at 25 °C in darkness, conidiomata black, acervular, gregarious or confluent, erumpent. Colony on MEA fast-growing, slightly raised, wooly, grayish-white with smooth dark sienna margin, reaching in 62.25 mm in diameter after 14 days at 25 °C in darkness, conidiomata black, gregarious or confluent. Colony on OA, fast-growing, slightly raised, wooly, white with smooth olivaceous buff margin, reaching 78.75 mm diameter after 14 days at 25 °C in darkness.

Ecology and host characteristics: Isolates L105, L106 (=CBS 149021), and L240 (=CBS 149020) belonging to the present species were recovered from vines of the indigenous wine grape cvs. Xynisteri and Giannoudi. Isolate L105 was co-isolated with *Ph. chlamydospora*.

Distribution: Cyprus, Iran, and New Zealand (*V. vinifera*), Latvia (*Rhododendron*), the Netherlands (*Pyrus communis* and *Rubus fruticosus*), and Italy (*Rosa canina* and *Quercus ilex*).

Notes: Multigene, as well as single locus phylogeny of LSU, *bt2*, and *tef1-a* cluster ex-type and *Spo. rosigena* isolates were collected in this study in a highly supported clade. The spore dimensions and all other conidial characteristics of the obtained isolates are in the same range to the ex-type (MFLU 15-0782).

### 2.4. Pathogenicity

**Figure 7:**
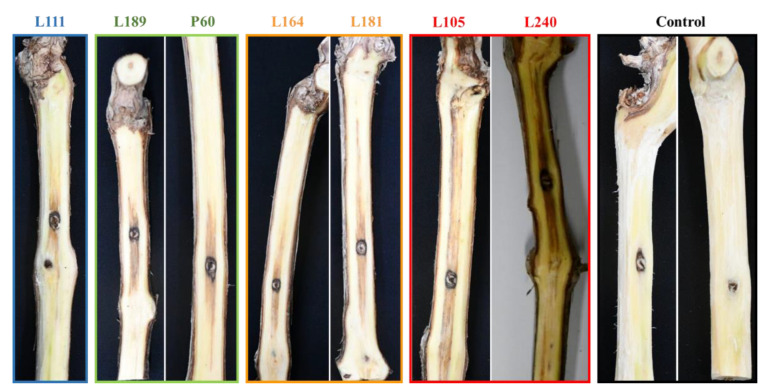
Pathogenicity of *Sporocadaceae* isolates collected in Cyprus vineyards on 2-year-old vines (cv. Xynisteri). Wood discoloration caused on lignified canes by isolates **L111**: *Seimatosporium cyprium*; **L189** and **P60**: *Sei. vitis-viniferae*; **L164** and **L181**: *Sporocadus kurdistanicus*; **L105** and **L240**: *Spo. rosigena* 12 months after inoculation. The control (non-inoculated) vines exhibit no disease symptoms.

**Figure 8:**
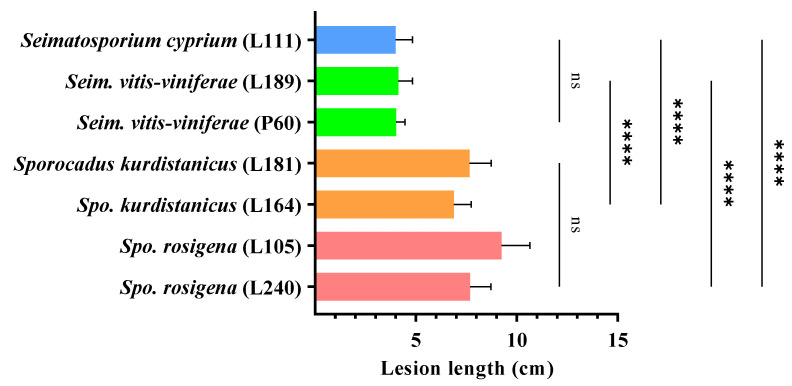
Mean lesion lengths caused 12 months post-inoculation by *Sporocadaceae* isolates on 2-year-old vines (cv. Xynisteri) during pathogenicity assays under field conditions. Each bar represents an individual tested isolate (n = 10), and vertical error bars indicate the corresponding standard deviation. Asterisks (****) and ns indicate the statistically significant (*p* < 0.0001) and non-significant differences (*p* < 0.05), respectively, following the analysis of variance and Tukey’s mean separation test procedures.

After a 12-month incubation period, all *Seimatosporium* and *Sporocadus* isolates evaluated were pathogenic to 2-year-old cv. Xynisteri potted grapevines. Dark brown to black vascular discoloration developed on the wood tissue below the bark, extending upward and downward from the point of inoculation, with the mean lengths shown in Figure 7, while foliar symptoms were absent in all treatments. Although all fungal species caused lesions, the virulence varied among the genera and species, with all *Sporocadus* isolates being more aggressive than *Seimatosporium* (*p* < 0.0001; Figure 8). More specifically, there was no significant difference in wood discoloration among the *Sporocadus* isolates, ranging from 9.24 ± 1.40 to 6.9 ± 0.85 cm (0.103 = *p* > 0.999). Similarly, the aggressiveness of *Seim. vitifusiforme* and *Seim. vitis-viniferae* isolates were not significantly different (*p* > 0.999), causing lesion lengths from 4.00 ± 0.83 to 4.13 ± 0.71 cm (Figure 8). No symptoms were evident in the non-inoculated (negative control) plants; thus, they were excluded from the statistical analyses. Successful re-isolations were made only from the inoculated vines and the recovery percentages ranged from 35–67% for *Seim*. *vitis-viniferae*, 40% for *Seim. cyprium*, 25–32% for *Spo. rosigena*, and 28–44% for *Spo. kurdistanicus*. Retrieved isolates were confirmed with those used in grapevine inoculations based on morphology (culture and conidial characteristics). Furthermore, no fungal isolates were obtained from the negative-control plants.

## 3. Discussion

This is the first study to explore the diversity and pathogenicity of *Sporocadaceae* species isolated from grapevines with GTD-related symptoms in the Mediterranean country of Cyprus. Four species of *Sporocadaceae* were identified by incorporating reference type and non-type sequences in multigene phylogenetic analyses. *Seim. cyprium* is a newly described and typified species, whereas two species in the genus of *Sporocadus*: *Spo. kurdistanicus* and *Spo. rosigena* constitute new reports from Europe on grapevines. Furthermore, *Seim. vitis-viniferae* is a new report from Cyprus.

Conidial morphology has been extensively used to dissect coelomyetous genera [35,48]; however, phylogenetic relationships at the species level have been refined in combination with sequence data analyses [31,32,33,50]. Nevertheless, until recently, the delineation of phylogenetic lineages in *Sporocadaceae* was still ambiguous to a certain extent, especially due to the lack of ex-type-sequence data in public repositories and designated epitypes, as well as the deficit of more-informative loci, such as partial protein-coding regions [26,34,38].

Phylogenetic analysis based on ITS and LSU sequences grouped the collected pestalotioid isolates in the genera *Seimatosporium* and *Sporocadus*. However, *tef1-a* phylogeny, as well as concatenated DNA sequence datasets (ITS, LSU, *bt2*, and *tef1-a*) combined with conidial phenology were sufficient for the species identification of obtained isolates as *Seim. cyrpium*, *Seim. vitis-viniferae*, *Spo*. *kurdistanicus*, and *Spo. rosigena*.

A combined four-loci phylogenetic analysis showed that *Seim. cyprium* formed an independent, fully supported clade that was phylogenetically distinct from *Seim. marivanicum* (CBS 143781), *Seim. luteosporum* (CBS142599) and *Seim. vitifusiforme* (CBS 142600). *Seim. cyprium* can be also distinguished from the phylogenetically most closely related species *Seim. marivanicum* by distinct conidial morphology (conidial dimensions, number of septa, morphology, and dimensions of conidial appendages), thus it is identified as a new species. Sexual fruiting structures of *Seim. cyprium* were also produced and described in in vitro homothallic pairings of collected isolates.

All available ITS LSU, *tub2* and *tef1-a* sequence data in the NCBI database, as well as those obtained presently, do not support species differentiation of *Seim. vitis* and *Seim. vitis-viniferae*, suggesting that their phylogenetic delineation is erratic. However, based on the conidial morphology of the ex-type cultures of *Seim. vitis* (MFLUCC 14-0051) and *Seim. vitis-viniferae* (CBS 123004), the two species can be clearly distinguished, suggesting that the most found *Sporocadacea* species in Cyprus is considered as *Seim. vitis-viniferae*. Previous studies [23,38,51] have described *Seim. vitis* in Iran and the USA, although the conidial descriptions provided do not match that of the ex-type specimen of the species, being closer to *Seim. vitis-viniferae* [31,34]. Furthermore, another report from Hungary has tentatively identified *Seim. vitis* based solely on ITS data [52], while a single study from Italy [53] reports *Seim. vitis* (dimensions are not provided) associated with grapevine states that the conidial morphology of the collected isolates matched the ex-type provided. Due to the abovementioned ambiguities, we recommend that the *bt2* and *tef1-a* sequence data of the *Seim. vitis* ex-type should be retrieved and analyzed, while its conidial description should be also reassessed.

Two non-appendaged *Sporocadaceae* species in the genus of *Sporocadus* associated with GTDs were also recorded in our study. *Spo. kurdistanicus* and *Spo. rosigena* isolates from Cyprus formed distinct and fully supported clades (ML/MP = 100/100) that clustered with respective ex-type cultures CBS 143778 and CBS 182.50, while they also shared similar conidial characteristics.

*Seimatosporium* and *Sporocadus* consist predominately of endophytes and saprobes of woody plants [17,33,34,43], although some species are pathogens of different plant hosts, such as eucalypt, blackberry, and grapevine [13,54,55]. More specifically, out of the nine *Seimatosporium* species previously reported from diseased or dead grapevine wood worldwide, only five (*Seim. botan*, *Seim. luteosporum*, *Seim. vitifusiforme*, *Seim. vitis*, *Seim. vitis-viniferae*) have been evaluated in wood inoculations of intact grapevines to confirm pathogenicity and Koch’s postulates. *Seim. botan* was isolated in Chile from mature vines bearing symptoms resembling those of Botryosphaeria dieback and was reported to be pathogenic on detached green shoots and rooted plants [56]. Similarly, *Seim. luteosporum* and *Seim. vitifusiforme* were first reported by Lawrence et al. (2018) in California, USA, from mature grapevines exhibiting typical dieback-type trunk disease symptoms, but due to a lack of re-isolation from inoculated vines, their pathogenic status remains unclear [38]. However, Grinbergs et al. (2021), confirmed the pathogenicity of *Seim. vitifusiforme* in rooted Petit Shyrah plants in Chile [37]. *Seim. vitis* has currently been the most reported species of the genus, found to be associated with GTD symptoms in California, USA, Hungary, Iran, and Italy, causing lesions and wood discoloration in pathogenicity assays [23,38,53,54]. The pathogenicity of *Seim. vitis-viniferae* was recently confirmed in Italy causing lesions of up to 24.70 cm on 1-year-old canes in an open field experiment with an 8-month incubation period [13]. Currently, there is a lack of information for *Seim. hysterioides*, *Seim. lonicerae*, *Seim*. *marivanicum*, and *Seim.* *parasiticum*. Similarly, little information is available on the pathogenicity of *Sporocadus* species associated with *V. vinifera*. *Spo. kurdistanicus* was recently isolated from grapevines exhibiting trunk disease symptoms in the Kurdistan province of Iran; however, it was found to be non-pathogenic on > 10-year-old vines. Furthermore, *Spo. rosigena* was also detected in association with GTDs in New Zealand [57], *Spo. rhododendri* has been isolated from *V. vinifera* canes in Australia [27], while *Spo. lichenicola* has been reported to cause blackberry cane dieback in Serbia and Oregon, USA [56,58]; however, their pathogenicity on grapevines has not been assessed yet. Herein, we present the ability of four *Sporocadaceae* species to cause vascular necrosis on grapevines, thus, contributing to the expanding knowledge on the agriculturally important group of GTD-related pathogens. In pathogenicity assays, isolates of all species were able to infect and colonize, and were able to produce brown to black vascular discoloration. Both *Sporocadus* species (*Spo. kurdistanicus* and *Spo. rosigena*) were significantly more aggressive compared to the *Seimatosporium* (*Seim. cyprium* and *Seim. vitis-viniferae*), while there was no difference in aggressiveness among isolates of the same genus. High recoveries of all tested isolates from inoculated vines were also evident, confirming Koch’s postulates.

GTDs are a disease complex resulting from interactions between taxonomically unrelated fungi colonizing grapevine wood [1,6,38]. Although limited research has been conducted on endophytes in economically important crops, particularly on their roles as latent pathogens [59], it is evident that GTD pathogens are routinely isolated from apparently healthy vines and found to express relatively long latency times in disease development [6,60,61], suggesting that some of them are likely commensal endophytes and/or latent saprobes that may act as opportunistic pathogens triggered by stressful edaphoclimatic factors or colonization density shifts of the residing microbiome [10,21,62,63,64]. In the present study, *Seimatosporium* and *Sporocadus* isolates were retrieved from wood cankers either solely or in combination with other known trunk pathogens, such as *Ph. chlamydospora, P. minimum*, and *B. dothidea*, while they have also been recovered from asymptomatic vine tissues [11,12,43,65]. Recent studies, that investigated the effect of dual inoculations between *Sporocadaceae* from grapevines and known GTD pathogens, report synergistic, as well as antagonistic interactions, confirming their involvement in the GTD complex [38,65]. Since mixed fungal infections are commonly found in vineyards, it is critical to clarify the complex microbial networks and elucidate pathogenesis and symptom development [66]. Understanding GTDs’ complexity will help us to prolong the sustainability and profitability of grape production, via cultural (late or double pruning) or other plant protection practices (application of plant protection products on pruning woods and the use of healthy planting material) [5,6,38,66].

## 4. Materials and Methods

### 4.1. Sampling and Fungal Isolation

**Figure 9:**
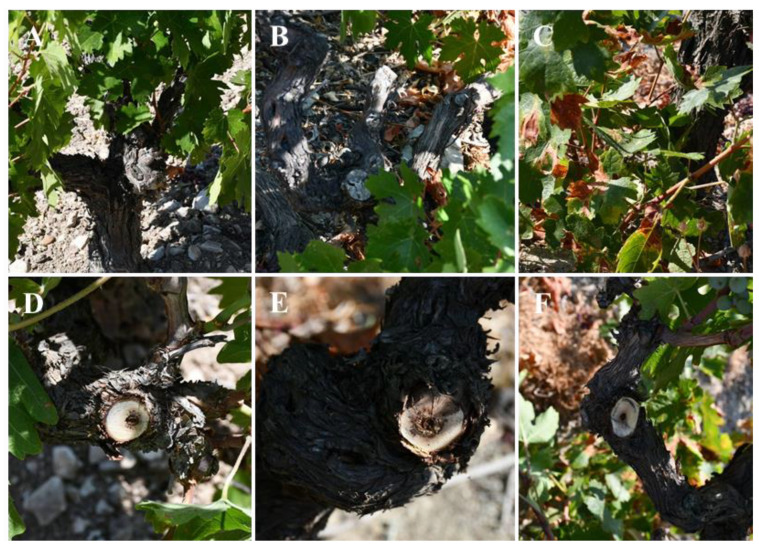
Symptoms observed on leaves, cordons, and trunks of grapevines (goblet-shaped) from isolations made from vineyards in Cyprus. (**A**,**B**) Dead spurs, and cordons. (**C**) Chlorotic and necrotic spots on leaves. (**D**–**F**) Trunk cross sections of vines (**A**–**C**) showing wedge-shape and brown-black streaks.

During 2017–2018, 10 vineyards in the provinces of Limassol and Paphos, Cyprus were surveyed and sampled for trunk diseases (Supplementary Table S2). Wood samples were collected from 3 vines per vineyard of the indigenous wine grape cvs. Giannoudi, Mavro, Promara, and Xynisteri exhibiting different typical GTD symptoms, including cankers, dead cordons and spurs, and tiger-stripe foliar symptoms (Figure 9). For fungal isolation wood, segments (1–2 cm thick) were cut off, debarked, washed in distilled water (dH_2_O), and fragmented in 4–5 pieces (5 mm thick). Accordingly, discolored wood pieces were disinfected in 95% ethyl alcohol for 1 min, rinsed with sterile dH_2_O, dried off in a laminar flow cabinet, and plated on potato dextrose Agar (PDA; HiMedia) amended with streptomycin sulfate (50 μg/mL). The plates were then incubated at 25 °C in darkness for up to 1–2 weeks and inspected daily to prevent the loss of slow-growing colonies from fast-growers. Hyphal tips of selected isolates were excised and transferred to fresh PDA plates at 25 °C to establish pure cultures that were maintained under the same conditions, and kept in an 40% aqueous glycerol solution in -80 °C. Based on colony characteristics, 16 *Sporocadaceae* isolates and isolates belonging to *Botryosphaeriaceae*, *Ph. chlamydospora*, and *Phaeoacremonium*, respectively, were used for morphological and molecular characterization [34,67,68].

### 4.2. DNA Isolation, PCR, and Sequencing

To avoid direct contact of the mycelium to the culture medium and its subsequent interference in the DNA extraction process, fungal cultures were grown on sterile cellophane discs (Sigma-Aldrich, St. Louis, MI, USA), which were placed on the top of the culture medium surface. Developed mycelia (approximately 14-day-old), were scraped off with a sterile spatula, lyophilized for 48 h, and homogenized with plastic micro-pestles into powder in the presence of liquid nitrogen. Total DNA was extracted according to Cary et al. (2009), following nanodrop quantification spectrophotometry [69]. The final DNA concentration of each isolate was adjusted to 20 ng/mL and stored at −20 °C for further use. The initial identification was based on sequences of ribosomal DNA fragments that included the ITS region. Furthermore, partial regions of the LSU, *tef1-a*, and *tub2* genes were amplified and sequenced for all *Sporocadaceae* isolates to elucidate their phylogenetic status. The sequence data of the *tub2* and *tef1-a* loci were used for species identification of the *Botryosphaeriaceae* and *Phaeoacremonium* isolates, while partial actin (*act*) gene data were also used for the latter group of isolates. PCR reactions were of 30-μL and performed using the KAPA Taq PCR kit (Sigma Aldrich, catalog no. BK1002) in a C1000TM Thermal Cycler (Bio-Rad). The PCR mixture contained 6 μL of KAPA Taq Buffer B (10×), 3 μL Mg^2+^, 0.6 μL of the dNTPs mixture (2.5 mmol/mL for each nucleotide), 1.5 μL of each primer (10 μM), 0.3 μL of *Taq* DNA polymerase (5 U/μL), 3 μL of DNA template (20 ng/μL), and 14.1 μL of sterile ddH_2_O. The primers used were ITS1 and ITS4 [70] for ITS, LROR [71] and Un-Lo28S1220 [72] for LSU, EF1-728F and EF1-986R [73] for *tef1-a*, T1 [74], Bt2b [75] for *tub2*, and ACT-512F and ACT-783R for *act* [73]. ITS amplification was performed using the following program: initial denaturation at 94 °C for 5 min, followed by 40 cycles of denaturation at 94 °C for 30 s, annealing at 57 °C for 30 s and extension at 72 °C for 1 min and a final extension step at 72 °C for 3 min. For LSU amplification, the n conditions were as follows: initial denaturation for 3 min at 95 °C; 35 cycles of denaturation for 30 s at 95 °C, primer annealing for 30 s at 57 °C, and extension for 1 min at 72 °C; and a final extension for 10 min at 72 °C. Cycling conditions for the rest of the loci were as described for LSU, but with at an annealing temperature of 58 °C for *act* and 60 °C for *tef1-a* and *tub2*, respectively. PCR amplicons were resolved on 1.5% agarose gels in Tris-acetate-EDTA buffer with a SYBR Safe DNA gel stain (Invitrogen, Carlsbad, CA, USA) and visualized under UV light. After confirmation by agarose gel electrophoresis, the PCR products were sequenced in both directions using the same primer pairs used for amplification by the Macrogen Europe B.V. (Amsterdam, the Netherlands).

### 4.3. Molecular Identifications and Phylogenetic Analysis

The isolates morphologically resembling typical trunk pathogens retrieved herein, such as *Ph. chlamydospora*, and the species belonging to *Botryosphaeriaceae* and *Phaeoacremonium,* were identified to the species level via BLASTn searches of the National Center for Biotechnology Information (NCBI), using respective sequences retrieved per taxon. Furthermore, after a literature review and NCBI BLASTn searches, ITS, LSU, *tub2*, and *tef1-a* sequences of 37 *Sporocadaceae* taxa (38 isolates) were retrieved and included in our phylogenetic analyses, along with the 16 *Sporocadaceaea* isolates obtained in this study (Table 3).

The consensus sequences of the four individual loci (ITS, LSU, *tub2*, and *tef1-a*) were aligned with MAFFT v. 7.490 [76] using default parameters, manually adjusted using Unipro Ugene v. 43.0 [77], and individual gene sequences were concatenated using SeqKit [78]. Single locus and concatenated alignments were then subjected to a maximum-likelihood (ML) analysis using IQtree version 2.0.3 [79]. The most appropriate substitution model was chosen based on the Bayesian Information Criterion (BIC) using the ModelFinder algorithm as implemented in IQtree version 2.0.3 [80]. Moreover, branch support was obtained using the bootstrap approximation option of IQtree [81], performing 1000 bootstrap replicates. Further support to the phylogenetic inference was provided by a maximum-parsimony (MP) analysis using MPBoot [82]. Similarly, to ML, the MP analysis was performed including 1000 bootstrap replicates. *Beltrania rhombica* (CBS 123.58) was selected as the outgroup taxon for both the ML and MP analyses (Table 3). The number of pairwise differences (PWD) between taxa on the concatenated sequence data was obtained using the distance estimation option implemented in MEGA X (Supplementary Table S1) [83]. New generated sequences were deposited in the NCBI GenBank database (Table 3). The resulting trees were edited in FigTree 1.4.4. The multigene sequences alignment and the trees obtained were deposited in the figshare repository (doi:10.6084/m9.figshare.20530188).

### 4.4. Morphological Description

*Sporocadaceae* isolates were morphologically described based on the colony, microscopic and stereoscopic characteristics of cultures grown on PDA, malt extract agar (MEA; Sigma-Aldrich), and oatmeal agar (OA; Sigma-Aldrich) at 25 °C for 2–4 weeks. Furthermore, 1-year-old lignified canes from the indigenous cv. Xynisteri were cut into 5 cm long segments and autoclaved twice (121 °C for 25 min, with a 24 h interval between autoclaves). The autoclaved segments were then placed in Petri dishes and immersed halfway in WA. Accordingly, three mycelial plugs from actively growing cultures were placed among the wood pieces, the cultures were incubated at 5 and 10 °C with a 12 h photoperiod and fruiting bodies formation was monitored weekly for up to 8 weeks [35]. Morphological observations of all reproductive structures were determined at appropriate magnifications using an Olympus BZX16 dissecting microscope and a Zeiss AX10 compound microscope, both equipped with color digital cameras Olympus ColorView I and Zeiss AxionCam MRc 5, respectively, and the minimum, maximum, mean, and standard deviation were calculated. The conidial length was measured from the base of the basal cell to the base of the apical appendage and the conidial width was measured to the widest point of the conidium. Furthermore, the conidial color, shape, and length of each conidial cell were also recorded. The colony morphology per culture medium was also described, while the colony colors were also rated per culture medium following Rayner’s (1970) charts [84].

### 4.5. Effect of Temperature on Mycelial Growth

Eight isolates belonging to four different *Sporocadaceae* species detected herein were randomly selected for the estimation of mycelial growth cardinal temperatures. Mycelial plugs (4 mm in diameter) from the margins of actively grown cultures were transferred into Petri dishes on PDA, MEA, and OA and incubated in the darkness from 5 to 30 °C at 5 °C intervals. Two perpendicular measurements of the diameter were recorded after 14 days. Three replicate plates were used per isolate and the experiment was repeated once. The optimum temperatures for mycelial growth and the maximum daily growth rate for each isolate were calculated based on regression curves of the temperature versus daily radial growth.

### 4.6. Pathogenicity Tests

In May 2020, a field trial was set up to evaluate the pathogenicity of seven isolates, representative of the *Seimatosporium* and *Sporocadus* species identified by phenotypic and phylogenetic analyses. Two-year-old vines of the cv. Xynisteri were grown in 10 L pots filled with potting mix (Miskaar; Lambrou Agro Ltd., Limassol, Cyprus) and amended with a slow-release fertilizer (Itapollina 12-5-15 SK; Lambrou Agro Ltd., Limassol, Cyprus). Lignified canes were aseptically wounded by drilling between the third and the fourth internode from their base. Subsequently, a 4 mm mycelial plug from 14-day-old cultures grown on PDA was placed in the wound that was then sealed with Vaseline (Unilever PMT Ltd., Nicosia, Cyprus) and wrapped with Parafilm (Sigma Aldrich) to prevent inoculum desiccation. Negative controls were inoculated with sterile PDA plugs. Five replicates were used per isolate, with an equal number of controls in a completely randomized design and the experiment was repeated once. All plants were placed under shade netting in open-field conditions and were drip-irrigated according to the weather 1–2 times per week for 30 min (0.5 L/h). Throughout the pathogenicity trial, the inoculated stems were routinely inspected for foliar symptoms, and 12 months after inoculation, were excised and transferred to the lab for further analyses. The newly developed green shoots and the bark were removed, and the length of the wood discoloration was measured in both directions from the inoculation point. The woody canes were washed in a sodium hypochlorite solution (5%) for 2 min and then rinsed twice with dH_2_O. Accordingly, 10 small, discolored wood pieces from each stem were disinfected in 95% ethanol for 1 min, and after drying, were placed in PDA dishes amended with streptomycin (50 mg/L) and chloramphenicol (50 mg/L). Plates were incubated at 25 °C in darkness for 1–2 weeks and the re-isolated colonies were identified morphologically.

### 4.7. Statistical Analyses

The effect of temperature on hyphal growth and wood discoloration length for each isolate were analyzed using an ANOVA to test for the normality and homogeneity of variances. Regression curves were fitted over different temperature treatments versus growth rate for each isolate. Data were analyzed using the Kruskal–Wallis test (non-parametric ANOVA), followed by Dunn’s posthoc multiple comparison test of means (*p* = 0.05). To assess the differences in the extent of the vascular discoloration induced by the different fungal isolates, pathogenicity data were log10 transformed to satisfy the normality requirements of the ANOVA. Bartlett’s test for homogeneity of variances was also performed for repeated experiments. Since variances were homogeneous (*p* < 0.05), the data were combined, and the means were compared with Tukey’s test at 5% of significance. All the statistical analyses were performed using SPSS (v. 25; IBM Corporation, New York, NY, USA).

## Figures and Tables

**Table 1:** Temperature-mycelial growth relationship for *Sporocadaceae* isolates from indigenous grapevine cultivars ^w^.

Species	Isolate	Adjusted Model ^x^					Optimum Temperature (°C) ^y^	Growth Rate (mm/day) ^z^
		*R* ^2^	*a*	*b*	*c*	*d*		
*Seimatosporium cyprium*	L111	0.98	−0.0003	0.0068	0.1964	−0.0451	24.1 a	4.4 cd
*Sei. cyprium*	L112	0.98	−0.0003	0.0065	0.2016	−0.0387	23.8 a	4.4 cd
*Sei. vitis-viniferae*	L34	0.98	−0.0009	0.0288	−0.0163	0.0867	21.1 c	4.1 e
*Sei. vitis-viniferae*	P60	0.99	−0.0006	0.0182	0.0785	−0.0383	22.2 b	4.1 e
*Sporocadus kurdistanicus*	L158	0.94	−0.0007	0.0201	0.0996	−0.0123	21.4 c	4.5 bc
*Spo. kurdistanicus*	L181	0.99	−0.0007	0.0192	0.0848	0.0306	20.3 d	3.8 f
*Spo*. *rosigena*	L105	0.99	−0.0007	0.0216	0.0826	−0.0557	22.2 b	4.8 a
*Spo. rosigena*	L240	0.99	−0.0007	0.0213	0.0849	−0.0446	22.1 b	4.7 ab

^w^ Data are the mean of six replicates per isolate. Means with the same letter, within a column, are not significantly (*p* = 0.05) different according to Kruskal–Wallis and the Dunn’s test for multiple comparisons. ^x^ Mycelial growth of PDA at 5 to 30 °C was adjusted to a quadratic model: *y* = *a*T^3^ + *b*T^2^ + *c*T + *d*, with *y* = mycelial growth (mm/day); *a*, *b*, *c*, *d* = regression coefficients; and *R*^2^ = coefficient of determination. ^y^ Optimum temperatures per isolate were estimated by the adjusted model. ^z^ Maximum growth rate per isolate was estimated by the adjusted model.

**Table 2:** Conidial features of *Seimatosporium* and *Sporocadus* species reported on *Vitis vinifera* ^a^.

Species	Conidia			Appendages			References
	Dimensions (μm)	Septa (No.)	L:W Ratio	Type	Apical (μm)	Basal (μm)	
*Seim. botan* (NBRC 104200 ^T^)	16–20 × 5–7 (av. 18 × 6) 16–20 × 4–5 (av. 18 × 4)	3 3	2.6–3.8 (av. 3) 4–5 (av. 4.6)	basal apical and basal	- 4–8 (av. 5.8)	4–8 (av. 6) 4–8 (av. 5.4)	[46]
*Seim. cyprium* (CBS 149019 ^T^)	15.7–23.1 × 3.9–5.3 (av. 18.5 × 4.5)	3	4.1	apical and basal	5.8–32.2 (av. 19.6)	2.6–29.0 (av. 16.5)	Present study
Seim. hysterioides *	12–16.5 × 5.2–6.5	3	-	often lacking, basal or both	10–15	10–15	[47]
Seim. lonicerae *	9–16 × 3.5–5 (av. 13 × 4.4)	3 (rarely 2)	3	both or only basal	3–7 (av. 5.5)	2–12 (av. 7)	[48]
*Seim. luteosporum* (CBS 142599 ^T^)	16.7–25.4 × 4.7–5.6 (av. 19.9 × 5.3)	3	-	apical and basal	10.1–24.2 (av. 17.9)	9.8–23.6 (av. 16.7)	[38]
*Seim. marivanicum* (CBS 143781 ^T^)	16–31 × 3–7 (av. 24 × 3.5)	3–6	5–6	apical and basal	7–20 (av. 15)	5–20 (av. 16)	[26]
Seim. parasiticum *	22–35 × 5–7 (av. 27.5 × 5.5)	3–5 (mostly 5)	5	apical and basal	2–5 (av. 3.5)	2–8 (av. 4.5)	[48]
*Seim. vitifusiforme* (CBS 142600 ^T^)	18.6–30.3 × 3.7–5.1 (av. 24.9 × 4.2)	3	-	apical and basal	7–12.6 (av. 10)	3.9–16.6 (av. 9.5)	[38]
*Seim. vitis* (MFLUCC 14-0051 ^T^)	34–40 × 14–17 (av. 37 × 15)	3	-	basal	-	4–8 (av. 5)	[31]
*Seim. vitis-viniferae* (CBS 123004 ^T^)	13.5–26 × 4.5–6 (av. 16.5 × 5.2)	3–6 (mostly 3)	3.2	basal or both	4–11 (av. 7)	4–10 (av. 7.9)	[34]
*Seim. vitis-viniferae* (CBS 149016)	13.5–18.6 × 4.6–5.8 (av. 16 × 5.2)	3–5 (mostly 3)	3.1	basal or both	2.5–24.1 (av. 11.2)	3.7–19.7 (av. 10.7)	Present study
*Spo. kurdistanicus* (CBS 143778 ^T^)	18–24 × 6.5–9.5 (av. 21.5 × 8)	3	3	absent	-	-	[26]
*Spo. kurdistanicus* (CBS 149022)	20.6–30.1 × 7.5–10 (av. 24.2 × 8.8)	3	2.8	absent	-	-	Present study
*Spo. lichenicola* (NBRC 32625)	18–25 × 5.5–8 (av. 21.6 × 7.2)	3 (rarely 5)	3	absent	-	-	[34]
Spo. macrospermus *	28–39 × 9–12.5	5	-	absent	-	-	[35]
Spo. rhododendri *	15.5–20 × 6.5–8.5	3	-	absent	-	-	[49]
*Spo. rosigena* (CBS 466.96)	12–14 × 5–7.5 (av. 13 × 6.5)	3 (rarely 2)	-	absent	-	-	[34]
*Spo. rosigena* (CBS 149021 ^T^)	13.2–17.9 × 5.3–6.8 (av. 15.4 × 6.2)	2–3 (mostly 3)	2.5	absent	-	-	Present study

^a^ CBS: Culture collection of the Westerdijk Fungal Biodiversity Institute, Utrecht, the Netherlands; NBRC: Biological Resource Center, National Institute of Technology and Evaluation, Chiba, Japan. T: ex-type strain. * Designated ex-type culture not available; - = not available or not applied.

**Table 3:** Isolates used in this study with details regarding their geographic origin, host, and GenBank accession numbers ^a^.

Species	Isolate ^c,d^	Country	Host	GenBank Accession Number ^b^			
				ITS	LSU	*tub2*	*tef1-a*
*Allelochaeta biseptata*	CBS 131116 ^ET^	Australia	*Eucalyptus oresbia*	MH554075	MH554286	MH554749	MH554510
*All. fusispora*	CBS 144172 ^T^	Australia	*Eucalyptus sp.*	MH554094	MH554304	MH554767	MH554528
*Beltrania rhombica*	CBS 123.58 ^T^	Mozambique	Mangrove swamp	MH857718	MH869260	MH704631	MH704606
*Botryosphaeria dothidea*	**P11**	**Cyprus**	***Vitis vinifera***	**ON679649**	…	**ON887280**	**ON887281**
*Diploceras hypericinum*	CBS 143885 ^ET^	Netherlands	*Hypericum perforatum*	MH554108	MH554316	MH554781	MH554542
*Phaeoacremonium minimum*	**L234**	**Cyprus**	***V. vinifera***	**ON679660**	…	**ON887279**	**ON887278**
*Phaeomoniella chlamydospora*	**LP262**	**Cyprus**	***V. vinifera***	**ON679662**	…	…	…
*Sarcostroma diversiseptatum*	CBS 189.81 ^T^	Australia	*Correa reflexa*	MH554016	MH554236	MH554692	MH554450
*Sar. grevilleae*	CBS 101.71 ^T^	Australia	*Grevillea rosmarinifolia*	MH553952	MH554175	MH554611	MH554370
*Sar. pestionis*	CBS 118153 ^T^	S. Africa	*Ischyrolepis cf. sieber*	DQ278923	DQ278925	MH554650	MH554408
*Seimatosporium botan*	NBRC 104200 ^T^	Japan	*Paeonia suffruticosa*	AB594799	AB593731	LC047770	…
*Seim. cyprium*	**L111 = CBS 149019**	**Cyprus**	***V. vinifera***	**ON680684**	**ON705769**	**ON695856**	**ON863790**
*Seim. cyprium*	**L112**	**Cyprus**	***V. vinifera***	**OΝ695889**	**ON692404**	**ON695848**	**ON863791**
*Seim. discosioides*	NBRC 104201	Japan	*Punica granatum*	AB594800	AB593732	LC047771	…
*Seim. germanicum*	CBS 437.87 ^T^	Germany	*…*	MH554047	MH554259	MH554723	MH554482
*Seim. luteosporum*	CBS142599 ^T^	USA	*V. vinifera*	KY706284	KY706309	KY706259	KY706334
*Seim. marivanicum*	CBS 143781 ^T^	Iran	*V. vinifera*	MW361952	MW361960	MW375352	MW375358
*Seim parasiticum*	NBRC 32682	Japan	*Physocarpus amurensis*	AB594808	AB593741	…	…
*Seim. physocarpi*	CBS 139968 ^T^	Russia	*Physocarpus opulifolius*	MH823022	MH823069	MH554676	MH554434
*Seim. pistaciae*	CBS 138865 ^T^	Iran	*Pistacia vera*	KP004463	KP004491	MH554674	MH554432
*Seim. rosae*	CBS 139823 ^ET^	Russia	*Rosa* sp.	LT853105	MH823070	LT853253	LT853203
*Seim. soli*	CBS 941.69 ^T^	Denmark	Soil (*Fagus sylvatica*)	MH554071	MH554282	…	MH554507
*Seim. vitifusiforme*	CBS 142600 ^T^	USA	*V. vinifera*	KY706296	KY706321	KY706271	KY706346
*Seim. vitis*	MFLUCC 14-0051 ^T^	Italy	*V. vinifera*	KR920363	KR920362	…	…
*Seim. vitis*	Napa764	USA	*V. vinifera*	KY706273	KY706298	KY706248	KY706323
*Seim. vitis-viniferae*	CBS 123004 ^T^	Spain	*V. vinifera*	MH553992	MH554211	MH554660	MH554418
*Seim. vitis-viniferae*	**P46 = CBS 149018**	**Cyprus**	***V. vinifera***	**ON679693**	**ON721334**	**ON695849**	**ON863776**
*Seim. vitis-viniferae*	**P56 = CBS 149017**	**Cyprus**	***V. vinifera***	**ON679694**	**ON721335**	**ON695850**	**ON863778**
*Seim. vitis-viniferae*	**P57**	**Cyprus**	***V. vinifera***	**ON680689**	**ON692397**	**ON695841**	**ON863779**
*Seim. vitis-viniferae*	**P60 = CBS 149016**	**Cyprus**	***V. vinifera***	**ON679695**	**ON721336**	**ON695851**	**ON863777**
*Seim. vitis-viniferae*	**P210**	**Cyprus**	***V. vinifera***	**ON680688**	**ON692398**	**ON695842**	**ON863780**
*Seim. vitis-viniferae*	**L** **3** **3**	**Cyprus**	***V. vinifera***	**ON680686**	**ON692399**	**ON695843**	**ON863782**
*Seim. vitis-viniferae*	**L34**	**Cyprus**	***V. vinifera***	**ON680687**	**ON692400**	**ON695844**	**ON863783**
*Seim. vitis-viniferae*	**L189**	**Cyprus**	***V. vinifera***	**ON680685**	**ON692401**	**ON695845**	**ON863781**
*Sporocadus biseptatus*	CBS 110324 ^T^	…	…	MH553956	MH554179	MH554615	MH554374
*Spo. cornicola*	CBS 143889	Germany	*Cornus sanguinea*	MH554121	MH554326	MH554794	MH554555
*Spo. cornii*	MFLUCC 14-0467 ^T^	Italy	*Cornus* sp.	KT162918	KR559739	…	…
*Spo. cotini*	CBS 139966 ^T^	Russia	*Cotinus coggygria*	MH554003	MH554222	MH554675	MH554433
*Spo. incanus*	CBS 123003 ^T^	Spain	*Prunus dulcis*	MH553991	MH554210	MH554659	MH554417
*Spo. italicus*	MFLUCC 14-1196 ^T^	Italy	*Crategus* sp.	MF614831	MF614829	…	…
*Spo. glandigenus*	NBRC 32677	Japan	*Fagus sylvatica*	AB594803	AB593735	…	…
*Spo. kurdistanicus*	CBS 143778 ^T^	Iran	*V. vinifera*	MW361950	MW361958	MW375350	MW375356
*Spo. kurdistanicus*	**L158 = CBS 149023**	**Cyprus**	***V. vinifera***	**ON695891**	**ON853905**	**ON695852**	**ON863784**
*Spo. kurdistanicus*	**L164**	**Cyprus**	***V. vinifera***	**ON695892**	**ON697292**	**ON695847**	**ON863786**
*Spo. kurdistanicus*	**L181 = CBS 149022**	**Cyprus**	***V. vinifera***	**ON695890**	**ON853906**	**ON695853**	**ON863785**
*Spo*. *lichenicola*	CPC 24528	Germany	*Juniperus communis*	MH554127	MH554332	MH554800	MH554562
*Spo*. *mali*	CBS 446.70 ^T^	Netherlands	*Malus sylvestris*	MH554049	MH554261	MH554725	MH554484
*Spo*. *microcyclus*	CBS 887.68	Netherlands	*Ribes* sp.	MH554068	MH554280	MH554744	MH554504
*Spo*. *multiseptatus*	CBS 143899 ^T^	Serbia	*Viburnum* sp.	MH554141	MH554343	MH554814	MH554576
*Spo. rosarum*	CBS 113832	Sweden	*Rosa canina*	MH553970	MH554189	MH554629	MH554388
*Spo*. *rosigena*	CBS 182.50	Netherlands	*Pyrus communis*	MH554013	MH554233	MH554689	MH554447
*Spo. rosigena*	**L** **105**	**Cyprus**	***V. vinifera***	**ON680683**	**ON692428**	**ON695846**	**ON863789**
*Spo. rosigena*	**L** **10** **6 = CBS 149021**	**Cyprus**	***V. vinifera***	**ON679667**	**ON853907**	**ON695854**	**ON863787**
*Spo. rosigena*	**L240 = CBS 149020**	**Cyprus**	***V. vinifera***	**ON679668**	**ON853908**	**ON695855**	**ON863788**
*Spo*. *rotundatus*	CBS 616.83 ^T^	Canada	*Arceuthobium pussilum*	MH554060	MH554273	MH554737	MH554496
*Spo*. *sorbi*	CBS 160.25 ^T^	…	…	MH554008	MH554229	MH554684	MH554442
*Spo*. *trimorphus*	CBS 114203 ^T^	Sweden	*R. canina*	MH553977	MH554196	MH554636	MH554395

^a^ GenBank accession numbers for the sequences of four loci: ribosomal DNA (rDNA) internal transcribed spacer region (ITS), rDNA large subunit (LSU), β-tubulin (*tub2*), and translation elongation factor 1-a (*tef1-a*) that were generated in this study (in bold) or from others. ^b^ Sequences from isolates in our collection are highlighted in bold. ^c^ CBS: Culture collection of the Westerdijk Fungal Biodiversity Institute, Utrecht, The Netherlands; CPC: Culture collection of Pedro Crous, housed at the Westerdijk Institute; MFLUCC: Mae Fah Luang University Culture Collection; NBRC: Biological Resource Center, National Institute of Technology and Evaluation, Chiba, Japan. ^d^ Status of the isolates = ET: ex-epitype; T: ex-type strain. Accession numbers in bold indicate isolates collected and characterized in the present study.

## Data Availability

Not applicable.

## Supplementary Materials

The following supporting information can be downloaded at: https://www.mdpi.com/article/10.3390/plants11202733/s1. Figure S1: Phylogenetic tree (log-likelihood: -4,806.893) resulting from the analysis of *tef1* from 47 *Sporocadaceae* isolates. Numbers represent maximum-likelihood/maximum-parsimony bootstrap values, respectively. Values represented by an asterisk were less than 70%. Scale bar represents the expected number of substitutions per site. The tree was rooted to *Beltrania rhombica* (CBS 123.58). Figure S2: Phylogenetic tree (log-likelihood: -7,026.633) resulting from the analysis of *tub2* from 48 *Sporocadaceae* isolates. Numbers represent maximum-likelihood/maximum-parsemony bootstrap values, respectively. Values represented by an asterisk were less than 70%. Scale bar represents the expected number of substitutions per site. The tree was rooted to *Beltrania rhombica* (CBS 123.58). Figure S3: Phylogenetic tree (log-likelihood: -1,641.838) resulting from the analysis of ITS from 54 *Sporocadaceae* isolates. Numbers represent maximum-likelihood/maximum-parsimony bootstrap values, respectively. Values represented by an asterisk were less than 70%. Scale bar represents the expected number of substitutions per site. The tree was rooted to *Beltrania rhombica* (CBS 123.58). Figure S4: Phylogenetic tree (log-likelihood: -1,744.611) resulting from the analysis of LSU from 54 *Sporocadaceae* isolates. Numbers represent maximum-likelihood/maximum-parsimony bootstrap values, respectively. Values represented by an asterisk were less than 70%. Scale bar represents the expected number of substitutions per site. The tree was rooted to *Beltrania rhombica* (CBS 123.58). Figure S5: Alignment of *Seimatosporium vitis* (Sv) and *Sei. vitis-viniferae* (Svv) *tef1-a* sequence data obtained in this study and currently available in the NCBI database. Base differences (substitutions: 8 and insertions/deletions: 2) are shown in color. Highlighted alignment position 252 depicts a T/C nucleotide substitution that is consisted among (Sv)/(Svv) isolates, respectively. All other nucleotide differences appear to be variably distributed between species. ^*^*tef1-a* sequence data of the (Svv) ex-type specimen (CBS 123004). This information is not available for the ex-type of (Sv) (MFLUCC 14-0051). Supplementary Table S1: Estimates of evolutionary divergence between concatenated sequence data used in the phylogenetic analysis of *Sporocadaceae*; Supplementary Table S2: Information on sampled vineyards in Cyprus.
