# Supplementary material for: A Novel Seimatosporium and Other Sporocadaceae Species Associated with Grapevine Trunk Diseases in Cyprus
Source: Plants (Basel). 2022 Oct 16;11(20):2733. doi: 10.3390/plants11202733 (PMC9608602; doi:10.3390/plants11202733)
Supplement: Supplementary file 1 [file plants-11-02733-s001.zip › Supplementary Figure S3_proofread.pdf]

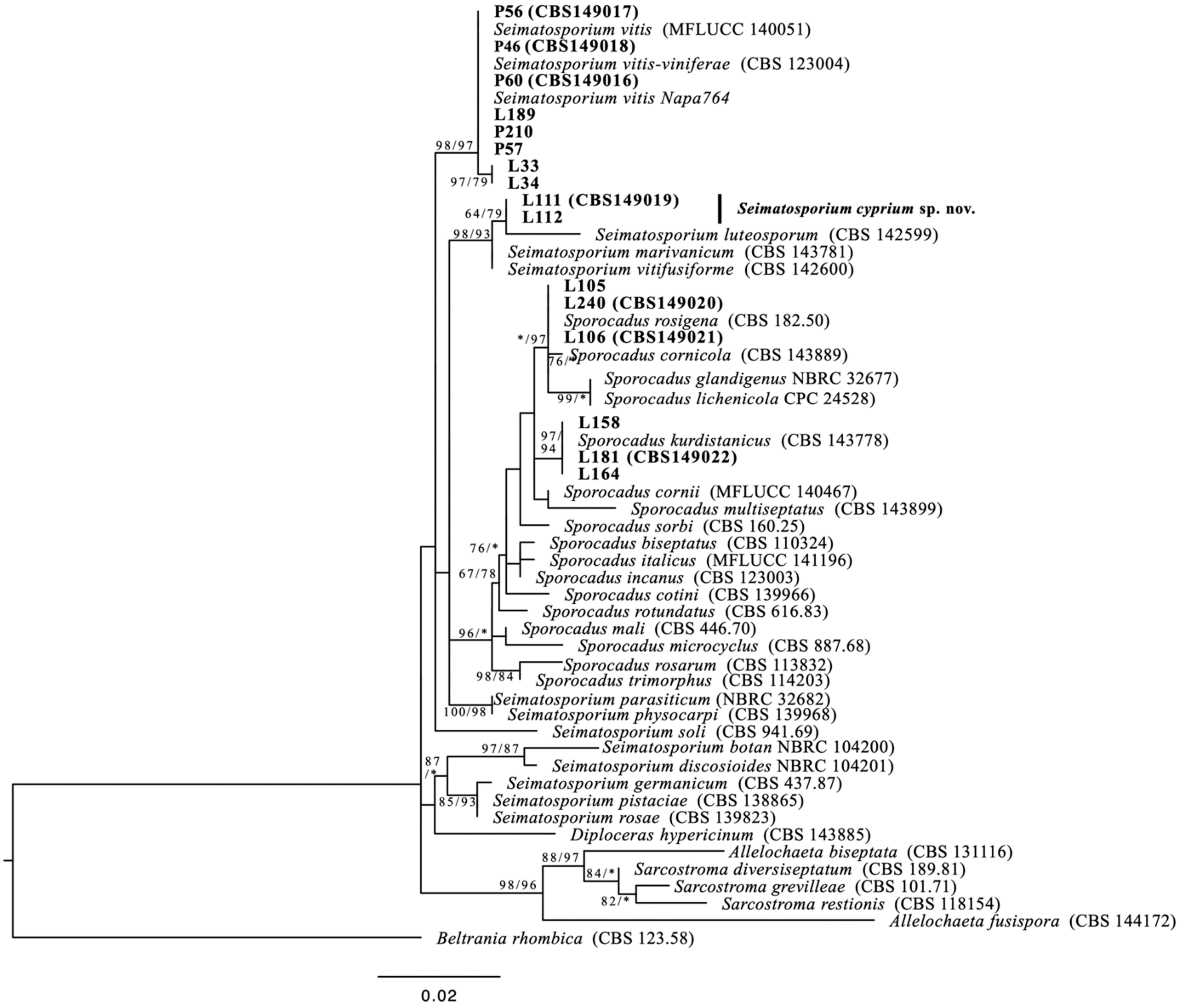

Supplementary Figure S3. Phylogenetic tree (log-likelihood: -1,641.838) resulting from the analysis of ITS from 54 *Sporocadaceae* isolates. Numbers represent maximum-likelihood/maximum-parsimony bootstrap values, respectively. Values represented by an asterisk were less than 70%. Scale bar represents the expected number of substitutions per site. The tree was rooted to *Beltrania rhombica* (CBS 123.58).
