# Supplementary material for: A Novel Seimatosporium and Other Sporocadaceae Species Associated with Grapevine Trunk Diseases in Cyprus
Source: Plants (Basel). 2022 Oct 16;11(20):2733. doi: 10.3390/plants11202733 (PMC9608602; doi:10.3390/plants11202733)
Supplement: Supplementary file 1 [file plants-11-02733-s001.zip › Supplementary Figure S5_proofread.pdf]

KY706330 (Sv)	AGTTTTGTCCTCACAGCCCTCACATCCACAACCATCAAATTTCCCATCTTGTCTGTTGCA	60
KY706327 (Sv)	AGTTTTGTCCTCACAGCCCTCACATCCACAACCATCAAATTTCCCATCTTGTCTGTTGCA	60
KY706326 (Sv)	AGTTTTGTCCTCACAGCCCTCACATCCACAACCATCAAATTTCCCATCTTGTCTGTTGCA	60
KY706329 (Sv)	AGTCTTGTCCTCACAGCCCTCACATCCACAACCATCAAATTTCCCATCTTGTCTGTTGCG	60
KY706324 (Sv)	AGTCTTGTCCTCACAGCCCTCACATCCACAACCAACAAATTTCCCATCTTGTCTGTTGCG	60
KY706332 (Sv)	AGTCTTGTCCTCACAGCCCTCACATCCACAACCATCAAATTTCCCATCT-GTCTGTTGCG	60
KY706331 (Sv)	AGTCTTGTCCTCACAGCCCTCACATCCACAACCATCAAATTTCCCATCTTGTCTGTTGCG	60
KY706328 (Sv)	AGTCTTGTCCTCACAGCCCTCACATCCACAACCATCAAATTTCCCATCTTGTCTGTTGCG	60
KY706325 (Sv)	AGTCTTGTCCTCACAGCCCTCACATCCACAACCATCAAATTTCCCATCTTGTCTGTTGCG	60
KY706323 (Sv)	AGTCTTGTCCTCACAGCCCTCACATCCACAACCATCAAATTTCCCATCTTGTCTGTTGCG	60
LS999505 (Sv)	AGTCTTGTCCTCACAGCCCTCACATCCACAACCATCAAATTTCCCATCTTGTCTGTTGCG	60
LS999504 (Sv	AGTCTTGTCCTCACAGCCCTCACATCCACAACCATCAAATTTCCCATCTTGTCTGTTGCG	60
LS999503 (Sv)	AGTCTTGTCCTCACAGCCCTCACATCCACAACCATCAAATTTCCCATCTTGTCTGTTGCG	60
LS999502 (Sv)	AGTCTTGTCCTCACAGCCCTCACATCCACAACCATCAAATTTCCCATCTTGTCTGTTGCG	60
MH554418 (Svv)*	AGTTTTGTCCTCACAGCCCTCACATCCACAACCATCAAATTTCCCATCTTGTCTGTTGCA	60
MH554402 (Svv)	AGTCTTGTCCTCACAGCCCTCACATCCACAACCATCAAATTTCCCATCTTGTCTGTTGCG	60
MN862448 (Svv)	AGTTTTGTCCTCACAGCCCTCACATCCACAACCATCAAATTTCCCATCTTGTCTGTTGCA	60
MN862451 (Svv)	AGTCTTGTCCTCACAGCCCTCACATCCACAACCATCAAATTTCCCATCTTGTCTGTTGCG	60
MN862450 (Svv)	AGTCTTGTCCTCACAGCCCTCACATCCACAACCATCAAATTTCCCATCTTGTCTGTTGCG	60
MN862447 (Svv)	AGTCTTGTCCTCACAGCCCTCACATCCACAACCATCAAATTTCCCATCTTGTCTGTTGCG	60
MN862446 (Svv)	AGTCTTGTCCTCACAGCCCTCACATCCACAACCATCAAATTTCCCATCTTGTCTGTTGCG	60
MN862449 (Svv)	AGTCTTGTCCTCACAGCCCTCACATCCACAACCATCAAATTTCCCATCTTGTCTGTTGCG	60
MN862445 (Svv)	AGTCTTGTCCTCACAGCCCTCACATCCACAACCATCAAATTTCCCATCTTGTCTGTTGCG	60
ON863777 (Svv)	AGTTTTGTCCTCACAGCCCTCACATCCACAACCATCAAATTTCCCATGTTGTCTGTTGCA	60
ON863778 (Svv)	AGTTTTGTCCTCACAGCCCTCACATCCACAACCATCAAATTTCCCATCTTGTCTGTTGCA	60
ON863779 (Svv)	AGTTTTGTCCTCACAGCCCTCACATCCACAACCATCAAATTTCCCATCTTGTCTGTTGCA	60
ON863780 (Svv)	AGTTTTGTCCTCACAGCCCTCACATCCACAACCATCAAATTTCCCATCTTGTCTGTTGCA	60
ON863776 (Svv)	AGTTTTGTCCTCACAGCCCTCACATCCACAACCATCAAATTTCCCATCTTGTCTGTTGCA	60
ON863781 (Svv)	AGTTTTGTCCTCACAGCCCTCACATCCACAACCATCAAATTTCCCATCTTGTCTGTTGCA	60
ON863782 (Svv)	AGTCTTGTCCTCACAGCCCTCACATCCACAACCATCAAATTTCCCATCTTGTCTGTTGCG	60
ON863783 (Svv)	AGTCTTGTCCTCACAGCCCTCACATCCACAACCATCAAATTTCCCATCTTGTCTGTTGCG	60

KY706330 (Sv)	GACAGAGTATTGCTCCATATCTATCTGCCTCCCG-CGTCCTGTGACGACGCGGGGCAATG	120
KY706327 (Sv)	GACAGAGTATTGCTCCATATCTATCTGCCTCCCG-CGTCCTGTGACGACGCGGGGCAATG	120
KY706326 (Sv)	GACAGAGTATTGCTCCATATCTATCTGCCTCCCG-CGTCCTGTGACGACGCGGGGCAATG	120
KY706329 (Sv)	GACAGAGTATTGCTCCATATCTATCTGCCTCCCGTCGTCCTGTGACGACGCGGGGCAATG	120
KY706324 (Sv)	GACAGAGTATTGCTCCATATCTATCTGCCTCCCGTCGTCCTGTGACGACGCGGGGCAATG	120
KY706332 (Sv)	GACAGAGTATTGCTCCATATCTATCTGCCTCCCGTCGTCCTGTGACGACGCGGGGCAATG	120
KY706331 (Sv)	GACAGAGTATTGCTCCATATCTATCTGCCTCCCGTCGTCCTGTGACGACGCGGGGCAATG	120
KY706328 (Sv)	GACAGAGTATTGCTCCATATCTATCTGCCTCCCGTCGTCCTGTGACGACGCGGGGCAATG	120
KY706325 (Sv)	GACAGAGTATTGCTCCATATCTATCTGCCTCCCGTCGTCCTGTGACGACGCGGGGCAATG	120
KY706323 (Sv)	GACAGAGTATTGCTCCATATCTATCTGCCTCCCGTCGTCCTGTGACGACGCGGGGCAATG	120
LS999505 (Sv)	GACAGAGTATTGCTCCATATCTATCTGCCTCCCGTCGTCCTGTGACGACGCGGGGCAATG	120
LS999504 (Sv)	GACAGAGTATTGCTCCATATCTATCTGCCTCCCGTCGTCCTGTGACGACGCGGGGCAATG	120
LS999503 (Sv)	GACAGAGTATTGCTCCATATCTATCTGCCTCCCGTCGTCCTGTGACGACGCGGGGCAATG	120
LS999502 (Sv)	GACAGAGTATTGCTCCATATCTATCTGCCTCCCGTCGTCCTGTGACGACGCGGGGCAATG	120
MH554418 (Svv)*	GACAGAGTATTGCTCCATATCTATCTGCCTCCCG-CGTCCTGTGACGACGCGGGGCAATG	120
MH554402 (Svv)	GACAGAGTATTGCTCCATATCTATCTGCCTCCCGTCGTCCTGTGACGACGCGGGGCAATG	120
MN862448 (Svv)	GACAGAGTATTGCTCCATATCTATCTGCCTCCCG-CGTCCTGTGACGACGCGGGGCAATG	120
MN862451 (Svv)	GACAGAGTATTGCTCCATATCTATCTGCCTCCCGTCGTCCTGTGACGACGCGGGGCAATG	120
MN862450 (Svv)	GACAGAGTATTGCTCCATATCTATCTGCCTCCCGTCGTCCTGTGACGACGCGGGGCAATG	120
MN862447 (Svv)	GACAGAGTATTGCTCCATATCTATCTGCCTCCCGTCGTCCTGTGACGACGCGGGGCAATG	120
MN862446 (Svv)	GACAGAGTATTGCTCCATATCTATCTGCCTCCCGTCGTCCTGTGACGACGCGGGGCAATG	120
MN862449 (Svv)	GACAGAGTATTGCTCCATATCTATCTGCCTCCCGTCGTCCTGTGACGACGCGGGGCAATG	120
MN862445 (Svv)	GACAGAGTATTGCTCCATATCTATCTGCCTCCCGTCGTCCTGTGACGACGCGGGGCAATG	120
ON863777 (Svv)	GACAGAGTATTGCTCCATATCTATCTGCCTCCCG-CGTCCTGTGACGACGCGGGGCAATG	120
ON863778 (Svv)	GACAGAGTATTGCTCCATATCTATCTGCCTCCCG-CGTCCTGTGACGACGCGGGGCAATG	120
ON863779 (Svv)	GACAGAGTATTGCTCCATATCTATCTGCCTCCCG-CGTCCTGTGACGACGCGGGGCAATG	120
ON863780 (Svv)	GACAGAGTATTGCTCCATATCTATCTGCCTCCCG-CGTCCTGTGACGACGCGGGGCAATG	120
ON863776 (Svv)	GACAGAGTATTGCTCCATATCTATCTGCCTCCCG-CGTCCTGTGACGACGCGGGGCAATG	120
ON863781 (Svv)	GACAGAGTATTGCTCCATATCTATCTGCCTCCCG-CGTCCTGTGACGACGCGGGGCAATG	120
ON863782 (Svv)	GACAGAGTATTGCTCCATATCTATCTGCCTCCCGTCGTCCTGTGACGACGCGGGGCAATG	120

ON863783 (Svv)	GACAGAGTATTGCTCCATATCTATCTGCCTCCCGTCGTCCTGTGACGACGCGGGGCAATG	120
KY706330 (Sv)	AAAATTGTTACCCCTCCTTTCACAAAAAGTCGTGCACAGACCACGAACAACCCCACCGAC	180
KY706327 (Sv)	AAAATTGTTACCCCTCCTTTCACAAAAAGTCGTGCACAGACCACGAACAACCCCACCGAC	180
KY706326 (Sv)	AAAATTGTTACCCCTCCTTTCACAAAAAGTCGTGCACAGACCACGAACAACCCCACCGAC	180
KY706329 (Sv)	AAAATTGTTACCCCTCCTTTCATAAAAAGTCGTGCACAGACCACGAACAACCCCACCGAC	180
KY706324 (Sv)	AAAATTGTTACCCCTCCTTTCACAAAAAGTCGTGCACAGACCACGAACAACCCCACCGAC	180
KY706332 (Sv)	AAAATTGTTACCCCTCCTTTCACAAAAAGTCGTGCACAGACCACGAACAACCCCACCGAC	180
KY706331 (Sv)	AAAATTGTTACCCCTCCTTTCACAAAAAGTCGTGCACAGACCACGAACAACCCCACCGAC	180
KY706328 (Sv)	AAAATTGTTACCCCTCCTTTCACAAAAAGTCGTGCACAGACCACGAACAACCCCACCGAC	180
KY706325 (Sv)	AAAATTGTTACCCCTCCTTTCACAAAAAGTCGTGCACAGACCACGAACAACCCCACCGAC	180
KY706323 (Sv)	AAAATTGTTACCCCTCCTTTCACAAAAAGTCGTGCACAGACCACGAACAACCCCACCGAC	180
LS999505 (Sv)	AAAATTGTTACCCCTCCTTTCACAAAAAGTCGTGCACAGACCACGAACAACCCCACCGAC	180
LS999504 (Sv)	AAAATTGTTACCCCTCCTTTCACAAAAAGTCGTGCACAGACCACGAACAACCCCACCGAC	180
LS999503 (Sv)	AAAATTGTTACCCCTCCTTTCACAAAAAGTCGTGCACAGACCACGAACAACCCCACCGAC	180
LS999502 (Sv)	AAAATTGTTACCCCTCCTTTCACAAAAAGTCGTGCACAGACCACGAACAACCCCACCGAC	180
MH554418 (Svv)*	AAAATTGTTACCCCTCCTTTCACAAAAAGTCGTGCACAGACCACGAACAACCCCACCGAC	180
MH554402 (Svv)	AAAATTGTTACCCCTCCTTTCACAAAAAGTCGTGCACAGACCACGAACAACCCCACCGAC	180
MN862448 (Svv)	AAAATTGTTACCCCTCCTTTCACAAAAAGTCGTGCACAGACCACGAACAACCCCACCGAC	180
MN862451 (Svv)	AAAATTGTTACCCCTCCTTTCACAAAAAGTCGTGCACAGACCACGAACAACCCCACCGAC	180
MN862450 (Svv)	AAAATTGTTACCCCTCCTTTCACAAAAAGTCGTGCACAGACCACGAACAACCCCACCGAC	180
MN862447 (Svv)	AAAATTGTTACCCCTCCTTTCACAAAAAGTCGTGCACAGACCACGAACAACCCCACCGAC	180
MN862446 (Svv)	AAAATTGTTACCCCTCCTTTCACAAAAAGTCGTGCACAGACCACGAACAACCCCACCGAC	180
MN862449 (Svv)	AAAATTGTTACCCCTCCTTTCACAAAAAGTCGTGCACAGACCACGAACAACCCCACCGAC	180
MN862445 (Svv)	AAAATTGTTACCCCTCCTTTCACAAAAAGTCGTGCACAGACCACGAACAACCCCACCGAC	180
ON863777 (Svv)	AAAATTGTTACCCCTCCTTTCACAAAAAGTCGTGCACAGACCACGAACAACCCCACCGAC	180
ON863778 (Svv)	AAAATTGTTACCCCTCCTTTCACAAAAAGTCGTGCACAGACCACGAACAACCCCACCGAC	180
ON863779 (Svv)	AAAATTGTTACCCCTCCTTTCACAAAAAGTCGTGCACAGACCACGAACAACCCCACCGAC	180
ON863780 (Svv)	AAAATTGTTACCCCTCCTTTCACAAAAAGTCGTGCACAGACCACGAACAACCCCACCGAC	180
ON863776 (Svv)	AAAATTGTTACCCCTCCTTTCACAAAAAGTCGTGCACAGACCACGAACAACCCCACCGAC	180
ON863781 (Svv)	AAAATTGTTACCCCTCCTTTCACAAAAAGTCGTGCACAGACCACGAACAACCCCACCGAC	180
ON863782 (Svv)	AAAATTGTTACCCCTCCTTTCACAAAAAGTCGTGCACAGACCACGAACAACCCCACCGAC	180
ON863783 (Svv)	AAAATTGTTACCCCTCCTTTCACAAAAAGTCGTGCACAGACCACGAACAACCCCACCGAC	180
KY706330 (Sv)	ATGCCTCTTATCGCACGCCTGCCTCGAATTGATTACTGACCCTCACAAAGGAAGCCGCCG	240
KY706327 (Sv)	ATGCCTCTTATCGCACGCCTGCCTCGAATTGATTACTGACCCTCACAAAGGAAGCCGCCG	240
KY706326 (Sv)	ATGCCTCTTATCGCACGCCTGCCTCGAATTGATTACTGACCCTCACAAAGGAAGCCGCCG	240
KY706329 (Sv)	ATGCCTCTTATCGCACGCCTGCCTCGAATTGATTACTGACCCTCATAAAGGAAGCCGCCC	240
KY706324 (Sv)	ATGCCTCTTATCGCACGCCTGCCTCGAATTGATTACTGACCCTCACAAAGGAAGCCGCCG	240
KY706332 (Sv)	ATGCCTCTTATCGCACGCCTGCCTCGAATTGATTACTGACCCTCACAAAGGAAGCCGCCG	240
KY706331 (Sv)	ATGCCTCTTATCGCACGCCTGCCTCGAATTGATTACTGACCCTCACAAAGGAAGCCGCCG	240
KY706328 (Sv)	ATGCCTCTTATCGCACGCCTGCCTCGAATTGATTACTGACCCTCACAAAGGAAGCCGCCG	240
KY706325 (Sv)	ATGCCTCTTATCGCACGCCTGCCTCGAATTGATTACTGACCCTCACAAAGGAAGCCGCCG	240
KY706323 (Sv)	ATGCCTCTTATCGCACGCCTGCCTCGAATTGATTACTGACCCTCACAAAGGAAGCCGCCG	240
LS999505 (Sv)	ATGCCTCTTATCGCACGCCTGCCTCGAATTGATTACTGACCCTCACAAAGGAAGCCGCCG	240
LS999504 (Sv)	ATGCCTCTTATCGCACGCCTGCCTCGAATTGATTACTGACCCTCACAAAGGAAGCCGCCG	240
LS999503 (Sv)	ATGCCTCTTATCGCACGCCTGCCTCGAATTGATTACTGACCCTCACAAAGGAAGCCGCCG	240
LS999502 (Sv)	ATGCCTCTTATCGCACGCCTGCCTCGAATTGATTACTGACCCTCACAAAGGAAGCCGCCG	240
MH554418 (Svv)*	ATGCCTCTTATCGCACGCCTGCCTCGAATTGATTACTGACCCTCACAAAGGAAGCCGCCG	240
MH554402 (Svv)	ATGCCTCTTATCGCACGCCTGCCTCGAATTGATTACTGACCCTCACAAAGGAAGCCGCCG	240
MN862448 (Svv)	ATGCCTCTTATCGCACGCCTGCCTCGAATTGATTACTGACCCTCACAAAGGAAGCCGCCG	240
MN862451 (Svv)	ATGCCTCTTATCGCACGCCTGCCTCGAATTGATTACTGACCCTCACAAAGGAAGCCGCCG	240
MN862450 (Svv)	ATGCCTCTTATCGCACGCCTGCCTCGAATTGATTACTGACCCTCACAAAGGAAGCCGCCG	240
MN862447 (Svv)	ATGCCTCTTATCGCACGCCTGCCTCGAATTGATTACTGACCCTCACAAAGGAAGCCGCCG	240
MN862446 (Svv)	ATGCCTCTTATCGCACGCCTGCCTCGAATTGATTACTGACCCTCACAAAGGAAGCCGCCG	240
MN862449 (Svv)	ATGCCTCTTATCGCACGCCTGCCTCGAATTGATTACTGACCCTCATAAAGGAAGCCGCCG	240
MN862445 (Svv)	ATGCCTCTTATCGCACGCCTGCCTCGAATTGATTACTGACCCTCATAAAGGAAGCCGCCG	240
ON863777 (Svv)	ATGCCTCTTATCGCACGCCTGCCTCGAATTGATTACTGACCCTCACAAAGGAAGCCGCCG	240
ON863778 (Svv)	ATGCCTCTTATCGCACGCCTGCCTCGAATTGATTACTGACCCTCACAAAGGAAGCCGCCG	240
ON863779 (Svv)	ATGCCTCTTATCGCACGCCTGCCTCGAATTGATTACTGACCCTCACAAAGGAAGCCGCCG	240
ON863780 (Svv)	ATGCCTCTTATCGCACGCCTGCCTCGAATTGATTACTGACCCTCACAAAGGAAGCCGCCG	240
ON863776 (Svv)	ATGCCTCTTATCGCACGCCTGCCTCGAATTGATTACTGACCCTCACAAAGGAAGCCGCCG	240

ON863781 (Svv)	ATGCCTCTTATCGCACGCCTGCCTCGAATTGATTACTGACCCTCACAAAGGAAGCCGCCG	240
ON863782 (Svv)	ATGCCTCTTATCGCACGCCTGCCTCGAATTGATTACTGACCCTCACAAAGGAAGCCGCCG	240
ON863783 (Svv)	ATGCCTCTTATCGCACGCCTGCCTCGAATTGATTACTGACCCTCACAAAGGAAGCCGCCG	240

KY706330 (Sv)	AACTCGGTAAGGGTTCTTTCAA	262
KY706327 (Sv)	AACTCGGTAAGGGTTCTTTCAA	262
KY706326 (Sv)	AACTCGGTAAGGGTTCTTTCAA	262
KY706329 (Sv)	AACTCGGTAAGGGTTCTTTCAA	262
KY706324 (Sv)	AACTCGGTAAGGGTTCTTTCAA	262
KY706332 (Sv)	AACTCGGTAAGGGTTCTTTCAA	262
KY706331 (Sv)	AACTCGGTAAGGGTTCTTTCAA	262
KY706328 (Sv)	AACTCGGTAAGGGTTCTTTCAA	262
KY706325 (Sv)	AACTCGGTAAGGGTTCTTTCAA	262
KY706323 (Sv)	AACTCGGTAAGGGTTCTTTCAA	262
LS999505 (Sv)	AACTCGGTAAGGGTTCTTTCAA	262
LS999504 (Sv)	AACTCGGTAAGGGTTCTTTCAA	262
LS999503 (Sv)	AACTCGGTAAGGGTTCTTTCAA	262
LS999502 (Sv)	AACTCGGTAAGGGTTCTTTCAA	262
MH554418 (Svv)*	AACTCGGTAAGGGTTCCTTCAA	262
MH554402 (Svv)	AACTCGGTAAGGGTTCCTTCAA	262
MN862448 (Svv)	AACTCGGTAAGGGTTCCTTCAA	262
MN862451 (Svv)	AACTCGGTAAGGGTTCCTTCAA	262
MN862450 (Svv)	AACTCGGTAAGGGTTCCTTCAA	262
MN862447 (Svv)	AACTCGGTAAGGGTTCCTTCAA	262
MN862446 (Svv)	AACTCGGTAAGGGTTCCTTCAA	262
MN862449 (Svv)	AACTCGGTAAGGGTTCCTTCAA	262
MN862445 (Svv)	AACTCGGTAAGGGTTCCTTCAA	262
ON863777 (Svv)	AACTCGGTAAGGGTTCCTTCAA	262
ON863778 (Svv)	AACTCGGTAAGGGTTCCTTCAA	262
ON863779 (Svv)	AACTCGGTAAGGGTTCCTTCAA	262
ON863780 (Svv)	AACTCGGTAAGGGTTCCTTCAA	262
ON863776 (Svv)	AACTCGGTAAGGGTTCCTTCAA	262
ON863781 (Svv)	AACTCGGTAAGGGTTCCTTCAA	262
ON863782 (Svv)	AACTCGGTAAGGGTTCCTTCAA	262
ON863783 (Svv)	AACTCGGTAAGGGTTCCTTCAA	262

Supplementary Figure S5. Alignment of *Seimatosporium vitis* (Sv) and *Sei. vitis-viniferae* (Svv) *tef1-a* sequence data obtained in this study and currently available in the NCBI database. Base differences (substitutions: 8 and insertions/deletions: 2) are shown in color. Highlighted alignment position 252 depicts a T/C nucleotide substitution that is consisted among (Sv)/(Svv) isolates, respectively. All other nucleotide differences appear to be variably distributed between species. **tef1-a* sequence data of the (Svv) ex-type specimen (CBS 123004). This information is not available for the ex-type of (Sv) (MFLUCC 14-0051).
