# Supplementary material for: A Novel Seimatosporium and Other Sporocadaceae Species Associated with Grapevine Trunk Diseases in Cyprus
Source: Plants (Basel). 2022 Oct 16;11(20):2733. doi: 10.3390/plants11202733 (PMC9608602; doi:10.3390/plants11202733)
Supplement: Supplementary file 1 [file plants-11-02733-s001.zip › Supplementary Table S2_proofread.pdf]

Supplementary Table S2. Information on sampled vineyards in Cyprus.

#	Province	Location	Collection date	Vineyard			
				Coordinates		Cultivar	Age
1	Limassol	Agios Therapon	13/4/2018	34° 47' 46.3" N	32° 50' 08.8" E	Mavro	30
2	Limassol	Chandria	6/6/2017	34° 56' 26.1" N	32° 59' 49.7" E	Mavro	50
3	Limassol	Kalo Chorio	8/6/2017	34° 51' 27.6" N	33° 01' 07.8" E	Xynisteri	40
4	Limassol	Kyrerounda	13/4/2018	34° 56' 41.0" N	32° 59' 14.1" E	Mavro	60
5	Limassol	Malia	23/6/2017	34° 48' 58.1" N	32° 47' 07.8" E	Giannoudi	18
6	Limassol	Zoopigi	8/6/2017	34° 51' 56.4" N	33° 00' 57.3" E	Xynisteri	30
7	Paphos	Lapithou	19/5/2017	34° 53' 51.7" N	32° 36' 08.0" E	Promara	12
8	Paphos	Salamiou	20/4/3018	34° 50' 23.5" N	32° 41' 12.5" E	Mavro	40
9	Paphos	Statos	18/5/2017	34° 53' 24.9" N	32° 36' 53.2" E	Mavro	80
10	Paphos	Panagia	18/5/2017	34° 55' 04.0" N	32° 37' 36.5" E	Mavro	30
